# Transcript features alone enable accurate prediction and understanding of gene expression in *S. cerevisiae*

**DOI:** 10.1186/1471-2105-14-S15-S1

**Published:** 2013-10-15

**Authors:** Hadas Zur, Tamir Tuller

**Affiliations:** 1 Blavatnik School of Computer Science, Tel Aviv University, 69978, Israel; 2 Department of Biomedical Engineering, the Engineering Faculty, Tel Aviv University, 69978, Israel; 3 The Sagol School of Neuroscience, Tel-Aviv University, Tel-Aviv, 69978, Israel

## Abstract

**Background:**

Gene expression is a central process in all living organisms. Central questions in the field are related to the way the expression levels of genes are encoded in the transcripts and affect their evolution, and the potential to predict expression levels solely by transcript features. In this study we analyze *S. cerevisiae*, a model organism with the most abundant relevant cellular and genomic measurements, to evaluate the accuracy in which expression levels can be predicted by different parts of the transcript. To this end, we perform various types of regression analyses based on a total of 5323 features of the transcript. The main advantage of the proposed predictors over previous ones is related to the accurate and comprehensive definitions of the relevant transcript features, which are based on biophysical knowledge of the gene transcription and translation processes, their modeling and evolution.

**Results:**

Cross validation analyses of our predictors demonstrate that they achieve a correlation of 0.68/0.68/0.70/0.61/0.81 with mRNA levels, ribosomal density, protein levels, proteins per mRNA molecule (PPR), and ribosomal load (RL) respectively (all p-values <$10^{-140}$). When we consider predictors that are based exclusively on the features related to different parts of the transcript (5'UTR, ORF, 3'UTR), the correlations with protein levels were 0.27/0.71/0.25 (all p-values <$10^{-5}$), suggesting that the information in the UTRs is redundant, and features of the ORF alone yield similar predictions to the ones obtained based on the entire transcript.

**Conclusions:**

The reported results demonstrate that in the analyzed model organism the expression levels of a gene are encoded in the transcript. Specifically, the prediction of a large fraction of the variance of the different gene expression steps based on transcript features alone is feasible in *S. cerevisiae*. We report dozens of novel transcript features related to expression levels predictions, demonstrating how such analyses can aid in understanding the gene expression process and its evolution, and how such predictors can be designed for other organisms in the future.

## Background

Gene expression is a fundamental cellular process by which proteins are synthesized based on the information coded in the genes. Understanding gene expression, and specifically how this process is encoded in the coding regions and UTRs and thus affects transcript evolution, has been the topic of dozens of papers in recent years [1-5]. The two major steps of gene expression are the transcription of the gene to mRNA molecules and the translation of mRNA molecules to proteins by the ribosome [6]. The protein abundance of a gene is related to its transcription rate/mRNA levels, its translation rate, and the degradation rate of the corresponding mRNA molecules and proteins. Specifically, if we assume constant mRNA levels, the translation rate should have a positive effect on the protein abundance, while the degradation rate should have a negative effect [7]. Expressly, it was suggested that protein abundance is correlated with adaptation to the tRNA pool [8], mRNA folding at the beginning of the ORF [9], ORF length [10], GC content [11], and various ancillary features of the 5'UTR [1]. In addition, it was found that highly expressed genes tend to evolve at a slower rate [12], and to have more protein-protein interactions [13].

In most of the biomedical studies, the protein levels of a gene are a far more important variable than its mRNA levels. However, today it is relatively easy to measure mRNA levels of genes [14], while for technical reasons the technologies for performing large scale measurements of protein abundance lag behind. For example, the GEO database includes hundreds of thousands of large scale measurements of mRNA levels, whilst there are only a few such large scale measurements of protein abundance [1,15-17]. Therefore, researchers from various fields are forced to use mRNA levels, the rather rough proxy of protein abundance, instead of the protein abundance itself. Thus, in recent years most of the studies in the field are aimed at predicting gene protein levels as opposed to mRNA levels. Concurrently, technologies to measure translation of mRNAs into proteins are now rapidly emerging, transforming our understanding of the proteome [1,9,15,17-31].

Previous studies aimed at predicting gene protein and mRNA levels are based on two major approaches, the machine learning approach and the biophysical approach. The biophysical approach is usually based on predictive simulations that are inspired by biophysical understanding of the studied processes. The machine learning approach, on the other hand, is based on statistical predictive inference of relations between sequence features and gene expression aspects, and it does not necessarily requires prior knowledge of the biophysical gene expression mechanisms.

Specifically, the first and more traditional machine learning approach includes, for example, codon composition features such as the Codon Adaptation Index (CAI, [32]), which is a simple effective measure of synonymous codon usage bias. The index uses a reference set of highly expressed genes from a species to assess the relative merits of each codon, and a score for a gene is calculated from the frequency of use of all codons in that gene. The index assesses the extent to which selection has been effective in modulating the pattern of codon usage. Other 'non biophysical' approaches include regressors and various machine learning techniques that are based on a combination of transcript sequence features and various large scale measurements related to gene expression [1,3,33].

The biophysical approach is based on physical understanding of the gene expression process, and includes computational biophysical models aimed at *simulating *the translation process and other stages of gene expression. Though theoretical physical models and simulations related to translation have been suggested over thirty years ago [34,35], only recently have such approaches been implemented on real large-scale genomic data. Biohysical models aim at considering the dynamics and physical nature of the process. The most basic features are the flow of ribosomes and the interactions between them [7,36-38]. These features can be modeled in a deterministic [38], or stochastic manner [7] in which the translation time of each codon is a random variable (*e.g*. with exponential distribution).

In this study we implemented, for the first time, a combined approach which employs the machine learning approach atop the biophysical one; in addition to the regular transcript features, various features and predictions that are outputs of the biophysical models are exploited and analyzed. We demonstrate the advantages of this approach over the previous ones.

**Figure 1:**
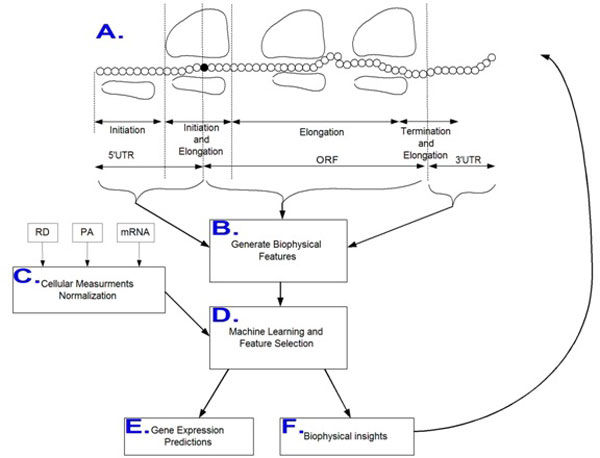
**A flow diagram and illustration of the study**. A-B. Based on a biophysical understanding of the translation process we extract features from the three parts of the transcript (5'UTR/ORF/3'UTR), corresponding to the three stages of translation (initiation, elongation, termination); but also to other stages in gene expression (transcription, mRNA and protein degradation), and from the entire transcript. C. Large scale cellular measurements of gene expression (mRNA, PA, RD) are collected and normalized. D.-F. Machine learning and feature selection approaches are employed based on the features and cellular data (D.) to infer predictors of gene expression (E. ) and improve the biophysical understanding of the gene expression process, its evolution, and its modeling (F.).

The major aims of this study are as follows (Figure 1): 1) Design accurate predictors of the protein levels, mRNA levels, proteins per mRNA molecule (PPR, see Additional file 1: Supplementary Methods), ribosomal load (RL, see Additional file 1: Supplementary Methods), and ribosomal densities of *S. cerevisiae *endogenous genes based *only *on features of its transcripts. 2) Report and understand the features with the highest contribution to these predictors. 3) Compare the contribution of the features in different parts of the transcript (5'UTR/ORF/3'UTR) to the expression levels of the gene, via the quality of the predictors based on each of these sets of features separately. 4) All the predictors inferred here are based *solely *on features of the transcript; in the strictest manner we ensured that no transcript feature is directly or indirectly based on gene expression measurements; this enables us to infer relations between properties of the transcripts and their expression aspects.

## Methods

All the details of the Methods appear in the Additional file 1 (Supplementary Methods).

## Results

To understand the effect of transcript features shaped by evolution on different stages of gene expression, we compare the contribution of each part of the transcript to protein abundance (PA), ribosomal density, mRNA levels, proteins per mRNA molecule (PPR) and the ribosomal load (RL), by building regression predictors for each segment, and for all the segments together. Focusing on the model organism *S. cerevisiae*, that has relatively ample and diverse large scale genome-wide data.

The evolutionary systems biology approach suggested in this study is novel for four main reasons. First, we generate for the first time a very large number of 5,323 transcript features related to computational biophysical modeling of the gene expression process; many of these features have been suggested and studied for the first time. Second, we propose and analyze here, for the first time, a combination of features related to the biophysical aspects of gene translation (and other stages of gene expression) via a machine learning approach. Third, we demonstrate how our approach can help to better predict variables related to gene expression, to rank different features, and to improve the understanding of the biophysics and evolution of gene expression. Finally, as aforementioned all the transcript features analyzed here are not based directly or indirectly on gene expression measurements, enabling accurate estimation of the fraction of gene expression variance that can be explained by the transcript, and the way it was shaped by evolution.

An illustration of the approach appears in Figure 1.

### Exploiting 5323 transcriptional features

The long list of features we extracted and analyzed, followed with explanations of their rationale appear in the Additional files 1, 2, 3. Briefly, we took into account amongst other features of the transcript, the lengths of the different segments, the ratios between the lengths of the UTRs and coding sequences and UTRs, number of ATGs, GC content, the predicted (MATLAB rnafold) and measured (PARS, [24]) folding energy in different parts of the transcript (Additional file 1: Supplementary Methods), the nucleotide context of the START codon [39]. In addition, it was shown that ATG codons near the beginning of the ORF may promote alternative translation initiation and thus should be under selection for elimination in highly expressed genes [40,41]; thus, we generated several features related to this phenomenon, such as the distance of the first alternative ATG from the main START codon, number of uORFs which are additional Open Reading Frames in the UTRs, what we termed sORFs which are shifted Open Reading Frames beginning at alternative ATGs downstream in the ORF from the main START codon, and the ATG context score [40] (Additional file 1: Supplementary Methods). To study the adaptation of codons to the tRNA pool we also considered the tAI [42] and the CAI, to estimate adaptations of the codons of highly expressed genes to various cellular resources [32]; to consider the effect of codon order and interactions between ribosomes on translation rates we consider the Totally Asymmetric Simple Exclusion Process (TASEP) translation rate prediction [43]. In addition, we considered the number of base pairs in the two dimensional folding of the mRNA in different parts of the transcript, measures of codon bias and amino acid bias (also taking into account the frequency of all codon and amino acid pairs) (see Additional files 1, 2, 3 for a detailed description, number and default value of the features in each predictor).

Features whose traditional estimation relies on expression levels were calculated in a novel manner independent of the expression levels, so that they are solely derived from the transcript (detailed description in the following section and Additional file 1: Supplementary Methods).

### Inferring families of predictors based on a robust Jackknifing procedure

We built linear and non linear predictors for the three parts of the transcript, the 5'UTR, ORF, and 3'UTR separately, and also combining the three together, in the following manner. The data was divided into terciles: a train, test and validation set, performing this sampling 100 times, thus resulting with 100 predictors per segment/entire transcript. The split between train and test helps avoiding over-fitting while repeating the procedure enables estimating the robustness of the inferred features. In addition, our approach shows that there is overlapping between the different features; hence, many predictors with similar performances exist. Our approach is similar but not identical to the random forest approach (see, for example [44], and a comparison in Additional file 1: Supplementary Methods ).

Additionally, this enabled us to perform statistical analyses of the prevalence, and thus significance of features. We implemented a greedy feature selection process, by which in each iteration every feature is added respectively to the growing regressor, and the feature contributing to the highest correlation is selected (Additional file 1: Supplementary Methods). At the end of each stage, the current predicted regressor coefficients of the selected features are assessed on the test set. The selected regressor is then evaluated on the validation set, in order to avoid overfitting.

The train set was utilized in-order to estimate features whose calculation relies on expression levels, instead of the highly expressed genes traditionally used for their estimation. These features include for example the CAI, tAI, TASEP and ATG Context Score (Additional file 1: Supplementary Methods). In order to deduce the contribution of expression levels via the optimization of such features to our regressor scheme's predictive power, we also compared the attained results to the ones obtained based on features that were estimated according to expression level measurements.

To model non-linear relations we used Multivariate Adaptive Regression Splines (MARS), which is a form of regression analysis introduced in [45]. It is a non-parametric regression technique, and can be seen as an extension of linear models that automatically models non-linearities and interactions. See Additional file 1 (Supplementary Methods) section for a detailed description of our predictors' methodology. The results of the non-linear predictors are similar to those of the linear predictors, providing an additional validation that the reported results are robust and are not specific to the (linear) model we chose to use here.

In each case mentioned above (gene expression measure, type of the regressor, and the way the features are inferred), we compute 100 predictors and report the performances of the median predictor (among the 100 ones) in terms of correlation with the real gene expression measurements; the features are ranked based on the number of times they appear in the different predictors (a score between 0 - 100).

Throughout the figure legends the following acronyms are used: × AA: × Amino Acid (*e.g*. C Amino Acid); XXX cod: XXX Codon (*e.g*. ACG Codon); BP: Base Pairs; ATG Dist: the distance of the first ATG in the relevant transcript segment (5'UTR/ORF/3'UTR) from the main start ATG; Best/Mean Rel ATG CS: The best or mean relative ATG Context Score (see Additional file 1: Supplementary Methods), if Rel is omitted then it refers to the absolute Context Score; 30C: first/last (if in the 5'UTR) 30 codons of the relevant segment; F0, F1, F2: we considered three reading frames, frame 0 is identical to the reading frame of the gene ORF, frames 1 and 2 represent a frame shift of 1 or 2 nucleotides relative to the main frame; FE: predicted folding energy (see Additional file 1: Supplementary Methods); Parallel Analysis of RNA Structure (PARS; see Additional file 1: Supplementary Methods): measured folding energy; expPARS: the exponent of the PARS score (see Additional file 1: Supplementary Methods).

### Predictors of gene expression variables based solely on features of the transcript

**Figure 2:**
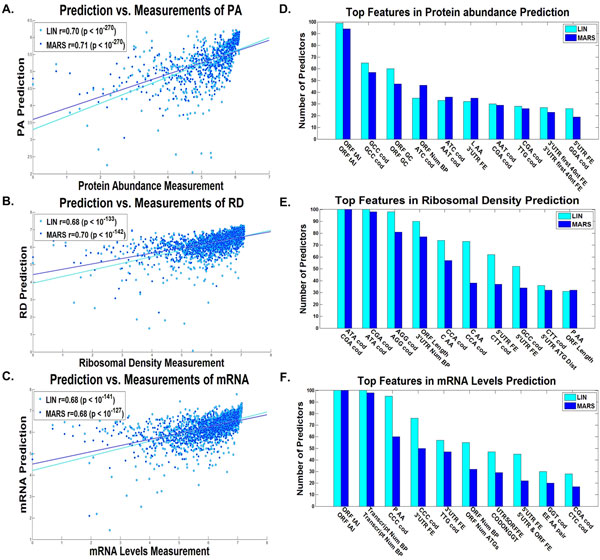
**Entire Transcript linear and non-linear predictors results**. Dot plot of the predictions *vs*. measurements for the validation set of the predictor with the median results for the A. protein levels, B. ribosomal densities, C. mRNA levels, for the entire transcript based on the, the combined linear (LIN) and non-linear (MARS) predictors (Additional file 1: Supplementary Methods). The best features according to the number of predictors they participated in (Additional file 1: Supplementary Methods) of the D. protein levels predictor, E. ribosomal density predictor, and F. mRNA levels predictor, for the entire transcript, for the combined linear (LIN) and non-linear (MARS) predictors (Additional file 1: Supplementary Methods).

**Figure 3:**
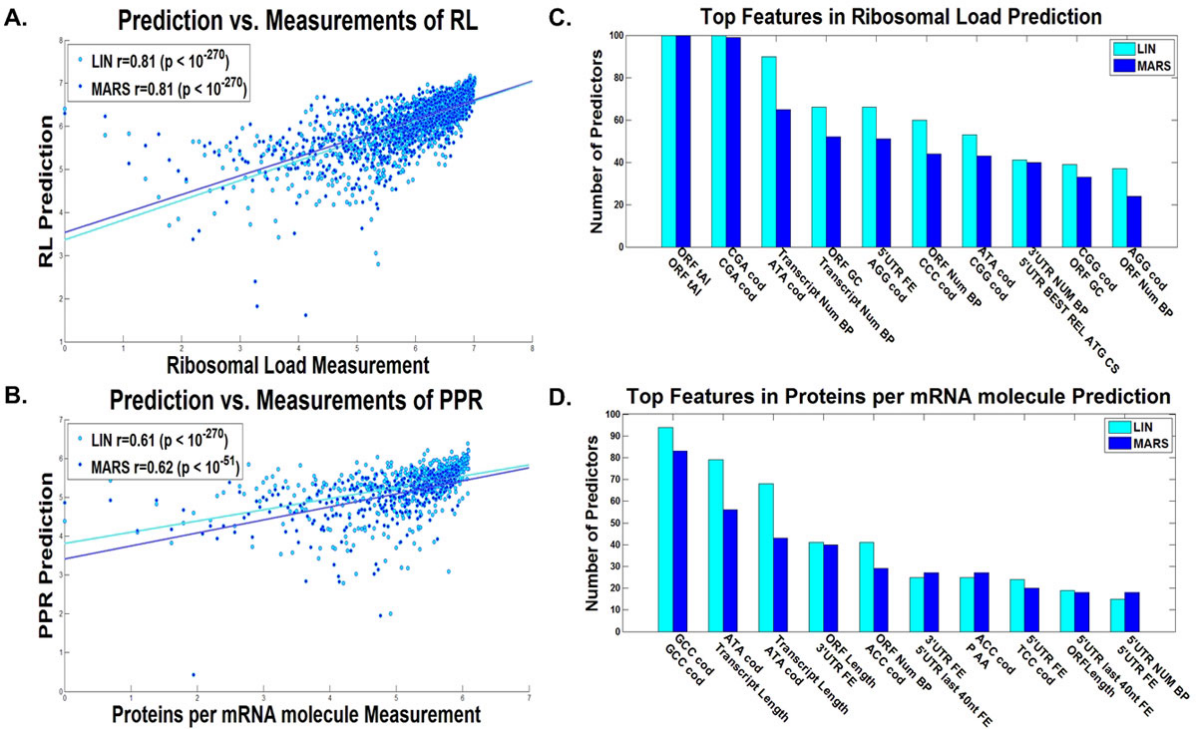
**Entire Transcript linear and non-linear predictors results**. Dot plot of the predictions *vs*. estimated measurements for the validation set of the predictor with the median results for the A. ribosomal load, and B. proteins per mRNA molecule, for the entire transcript, for the combined linear (LIN) and non-linear (MARS) predictors (Additional file 1: Supplementary Methods). The best features according to the number of predictors they participated in (Additional file 1: Supplementary Methods) of the C. ribosomal load predictor, and D. proteins per mRNA molecule predictor, for the entire transcript, for the combined linear (LIN) and non-linear (MARS) predictors (Additional file 1: Supplementary Methods).

At the first stage we investigated how well measures of gene expression can be predicted based on *all *the features of the transcript. To this end as aforementioned, for all the gene expression variables we repeatedly sampled a tercile of the data (train set), inferred greedily a predictor based on the transcript features, terminating its construction based on the second tercile (test set; Additional file 1: Supplementary Methods), and implemented it on the remainder of the data (validation set; Additional file 1: Supplementary Methods). Figure 2A-C includes the dot plot and correlation of the predicted *vs*. real protein levels (A), ribosomal densities (B), mRNA levels(C), and Figure 3A-B includes the dot plot and correlation of the predicted *vs*. estimated ribosomal load (A), and proteins per mRNA molecule (B), respectively, for the median linear and non-linear predictors (Additional file 1: Supplementary Methods). As can be seen in Figures 2, 3 for the linear/non-linear regressors, all the correlations are significantly high -- a correlation of 0.70/0.71 with protein levels (based on 20/10 features on average), 0.68/0.7 with ribosomal density (based on 24/11 features on average), 0.68/0.68 with mRNA levels (based on 22/12 features on average), 0.81/0.81 with ribosomal load (based on 24/13 features on average), and 0.61/0.62 with proteins per mRNA molecule (based on 18/11 features on average), (all p-values < 10^-270^), with all the predictors based on less than 24 features on average. These results are significantly higher than those previously reported for biophysical based models [7] or machine learning based models [3] alone (and when considering only transcript features). Specifically, the results demonstrate that variables related to the expression levels can be predicted with very high accuracy based on the transcript alone (correlation above 0.61 in all cases).

Figure 2D-F includes the top features used in many of the predictors (maximal value is 100, as the number of predictors built for each translation measure), for real protein levels (D), ribosomal density (E), and mRNA levels (F), and Figure 3C-D for the estimation of ribosomal load (C), and proteins per mRNA molecule (D) respectively. These features can elucidate the different mechanisms of gene expression, the way the efficiency of transcription and translation is encoded in the transcript, and the manner in which evolution shapes transcript sequences. The following is a brief set of examples:

One prominent feature is the tAI, which is based on the adaptation of codons to the tRNA pool of the organism [42]; as can be seen, tAI is a top feature in the case of mRNA, PA, and RL predictions. It was suggested that tAI, a measure of the adaption to the tRNA pool is higher in highly expressed genes due to stronger such selection in these genes [36,42,46]. Specifically the adaptation to the tRNA pool affects the translation elongation speed and thus improves the translation rate, hence effecting PA in a causal way [8] (a possible explanation for the observed contribution of this feature to PA prediction); in addition, it is known that there is a contrapositive relation between ribosomal speed and density [9,47]; thus, high translation elongation speed should decrease ribosomal density and therefore decrease the cost of protein expression in a non-causal way [9]; this relation is more important in genes with high mRNA levels and/or high ribosomal density that potentially consume more ribosomes (a possible explanation for the observed contribution of this feature to mRNA, PA, and RL predictions).

The strength of the folding along the different parts of the RNA transcript is also known to contribute to the efficiency of various gene expression steps, including translation initiation [8,9,48] and translation elongation [49,50]. Folding was also suggested to be under stronger selection (for strong folding) in highly expressed genes possibly to prevent aggregation of mRNA molecules [49]. Indeed, we see in all predictors (mRNA, RD, RL, PA, and PPR) variables related to the folding of the mRNA and its GC content in different parts of the transcript.

Another important feature that appears in the cases of RD and PPR prediction is the length of the ORF or transcript, supporting the conclusion that highly translated genes in yeast are under selection to be more compact (*e.g*. to minimize cellular resources such as the metabolic costs needed for their synthesis) [51].

Interestingly an important feature related to RD is the folding at the beginning of the 5'UTR, which is known to be related to the efficiency of translation initiation (strong folding decreases the efficiency of translation initiation [9,47]). In the case of mRNA levels, the folding and nucleotide composition of the 3'UTR are important features that may be related to the mRNA degradation rate [52,53].

Finally, a long list of codons and amino acids appears in the different predictors.

Among others, the frequency of the codons GGT and CTC appear in the mRNA predictor and tend to have negative coefficients, while codon CCC tends to have a positive coefficient. These codons can be related to mRNA levels in a causal way; for example, by increasing/decreasing transcription efficiency or effecting degradation rate. These codons may be related to mRNA levels in a non-causal way by having positive/negative effect on translation and since PA and mRNA levels tend to correlate.

Codons features tend to appear also in other predictors, for example, the codons CGA and ATA appear in the RD predictor and tend to have positive coefficients; the codons GCC and ATC tend to appear in the PA predictor with positive coefficients; the codons ATA and GCC tend to appear in the PPR predictor with positive coefficients; codons CGA and CCC tend appear in the RL predictor with negative coefficients.

As mentioned, the predictors also include features such as tAI that correspond to codon elongation rates; thus, this fact may suggest that these codons are not represented accurately in the current elongation rate measures (*e.g*. the tAI and CAI).

### Predictors based on the 5'UTR, ORF, and 3'UTR features separately

**Figure 4:**
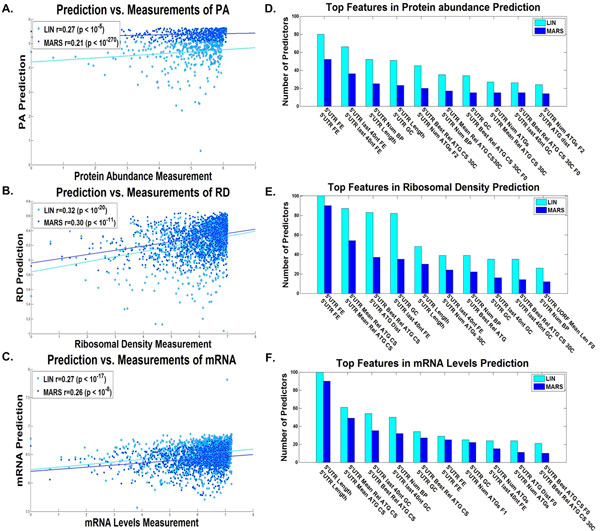
**5'UTR linear and non-linear predictors results**. Dot plot of the predictions *vs*. measurements for the validation set of the predictor with the median results for the A. protein levels, B. ribosomal densities, C. mRNA levels, for the 5'UTR linear (LIN) and non-linear (MARS) predictors (Additional file 1: Supplementary Methods). The best features according to the number of predictors they participated in (Additional file 1: Supplementary Methods) of the D. protein levels predictor, E. ribosomal density predictor, and F. mRNA levels predictor, for the 5'UTR linear (LIN) and non-linear (MARS) predictors (Additional file 1: Supplementary Methods).

**Figure 5:**
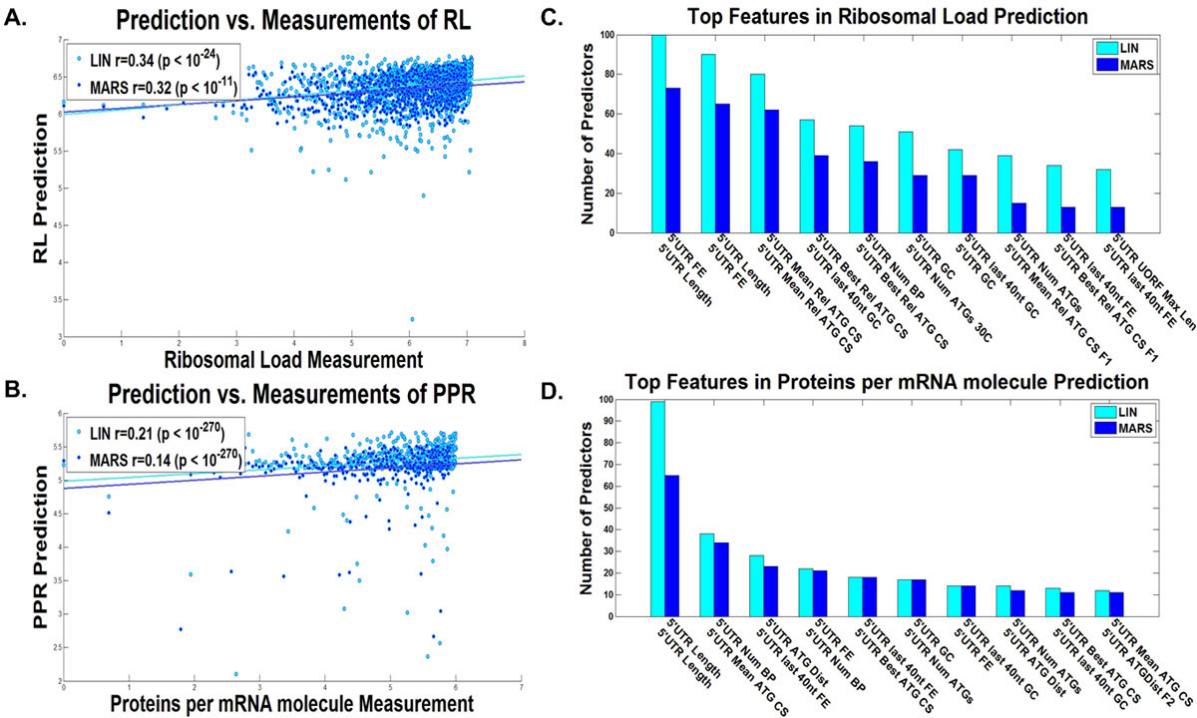
**5'UTR linear and non-linear predictors results**. Dot plot of the predictions *vs*. estimated measurements for the validation set of the predictor with the median results for the A. ribosomal load, and B. proteins per mRNA molecule, for the 5'UTR linear (LIN) and non-linear (MARS) predictors (Additional file 1: Supplementary Methods). The best features according to the number of predictors they participated in (Additional file 1: Supplementary Methods) of the C. ribosomal load predictor, and D. proteins per mRNA molecule predictor, for the 5'UTR linear (LIN) and non-linear (MARS) predictors (Additional file 1: Supplementary Methods).

**Figure 6:**
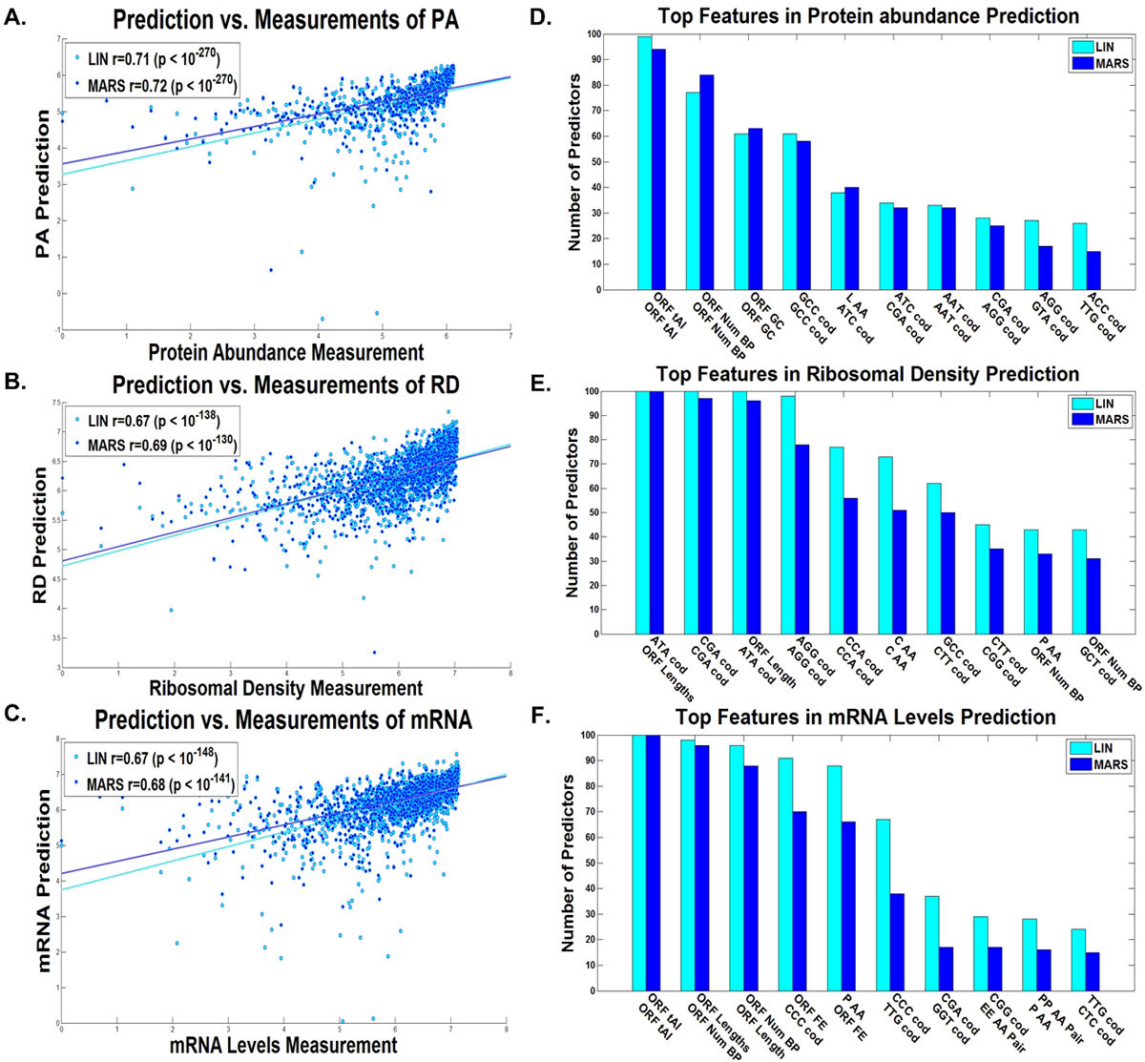
**ORF linear and non-linear predictors results**. Dot plot of the predictions *vs*. measurements for the validation set of the predictor with the median results for the A. protein levels, B. ribosomal densities, C. mRNA levels, for the ORF linear (LIN) and non-linear (MARS) predictors (Additional file 1: Supplementary Methods). The best features according to the number of predictors they participated in (Additional file 1: Supplementary Methods) of the D. protein levels predictor, E. ribosomal density predictor, and F. mRNA levels predictor, for the ORF linear (LIN) and non-linear (MARS) predictors (Additional file 1: Supplementary Methods).

**Figure 7:**
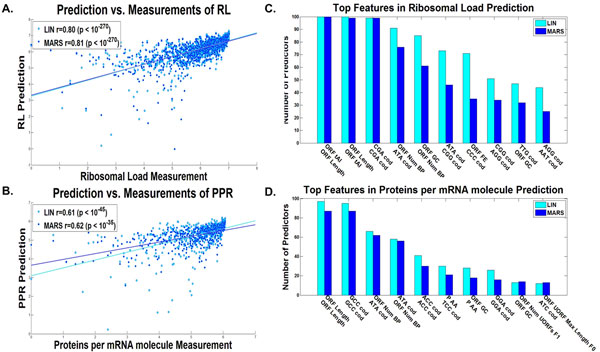
**ORF linear and non-linear predictors results**. Dot plot of the predictions *vs*. estimated measurements for the validation set of the predictor with the median results for the A. ribosomal load, and B. proteins per mRNA molecule, for the ORF linear (LIN) and non-linear (MARS)predictors (Additional file 1: Supplementary Methods). The best features according to the number of predictors they participated in (Additional file 1: Supplementary Methods) of the C. ribosomal load predictor, and D. proteins per mRNA molecule predictor, for the ORF linear (LIN) and non-linear (MARS) predictors (Additional file 1: Supplementary Methods).

**Figure 8:**
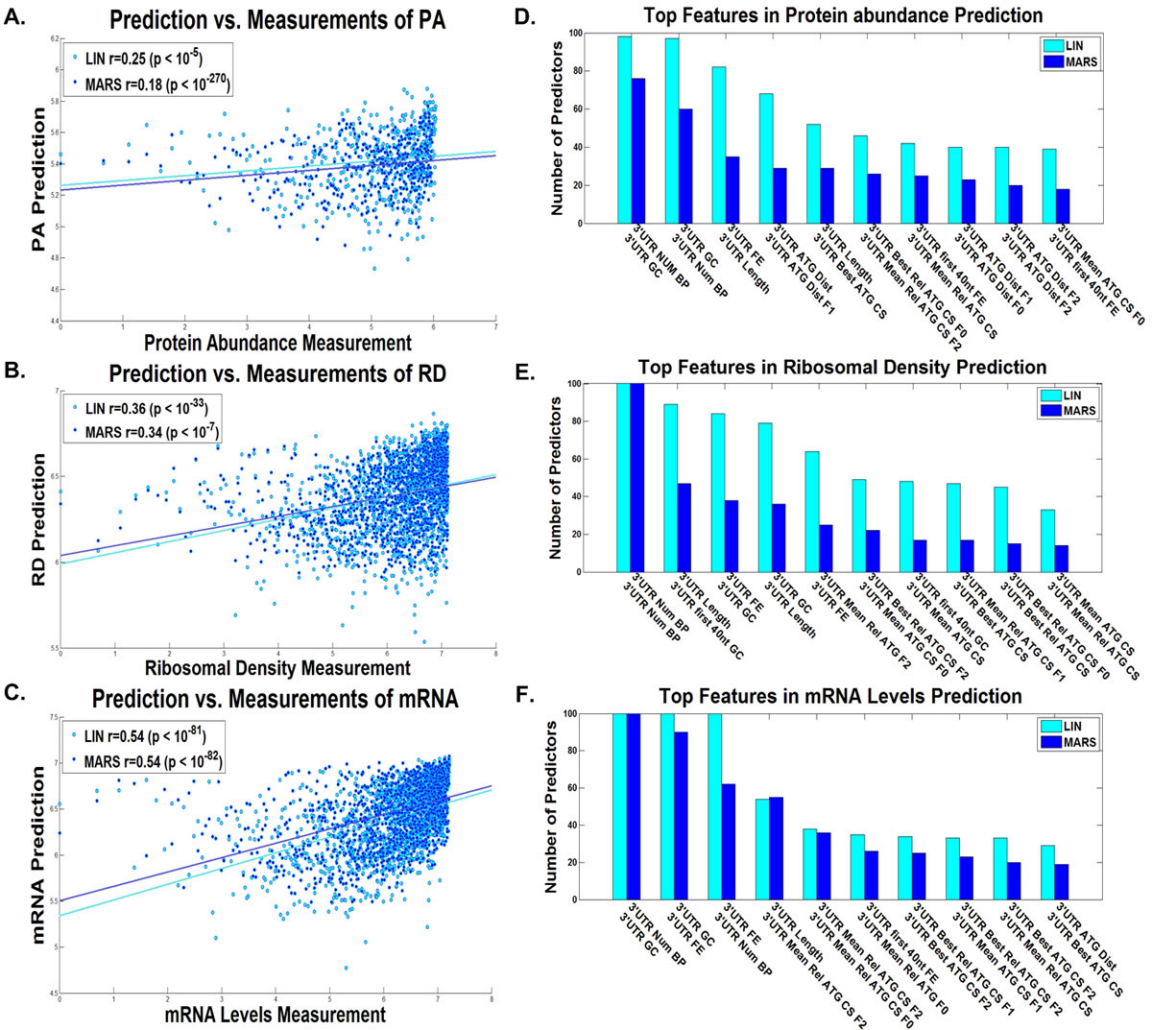
**3'UTR linear and non-linear predictors results**. Dot plot of the predictions *vs*. measurements for the validation set of the predictor with the median results for the A. protein levels, B. ribosomal densities, C. mRNA levels, for the 3'UTR linear (LIN) and non-linear (MARS) predictors (Additional file 1: Supplementary Methods). The best features according to the number of predictors they participated in (Additional file 1: Supplementary Methods) of the D. protein levels predictor, E. ribosomal density predictor, and F. mRNA levels predictor, for the 3'UTR linear (LIN) and non-linear (MARS) predictors (Additional file 1: Supplementary Methods).

**Figure 9:**
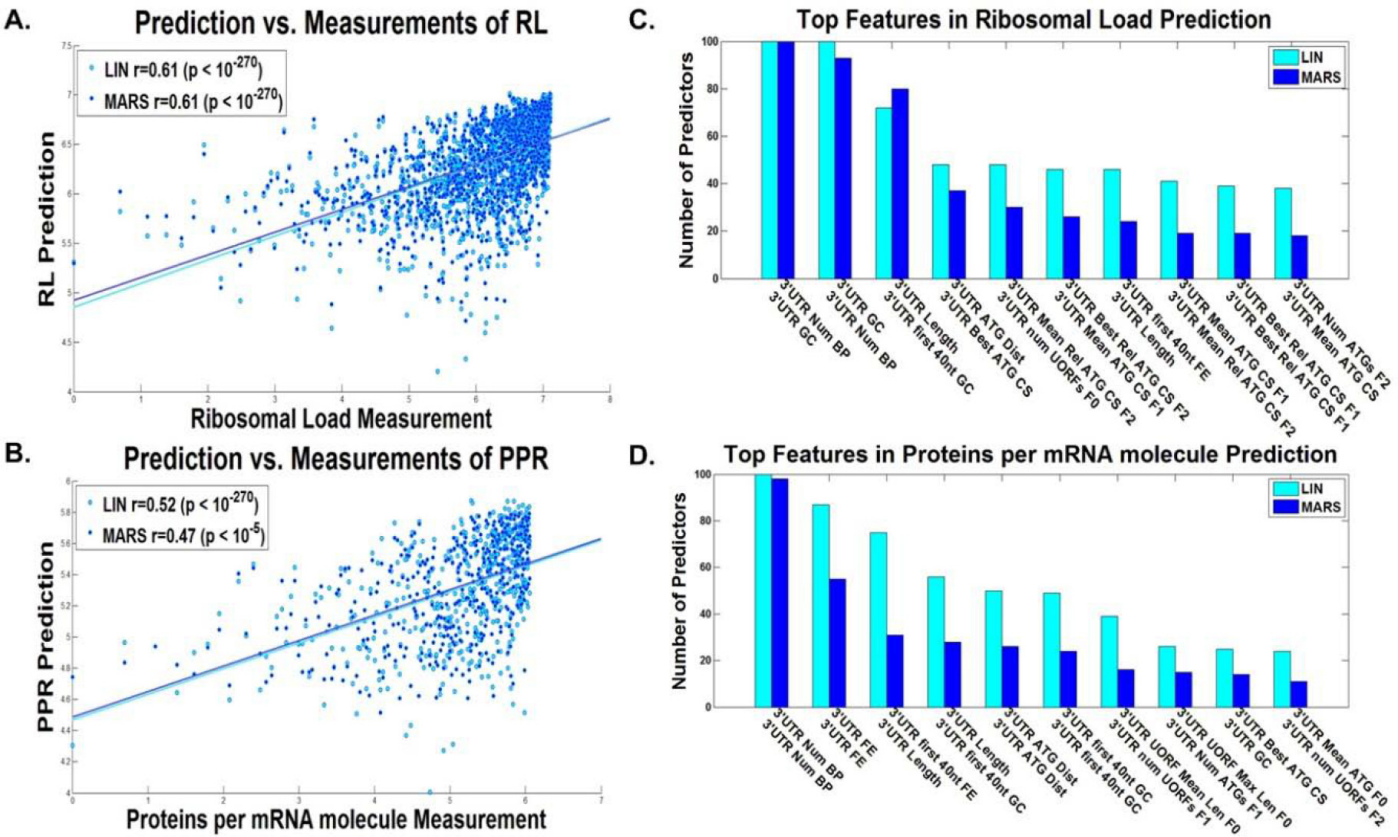
**3'UTR linear and non-linear predictors results**. Dot plot of the predictions *vs*. estimated measurements for the validation set of the predictor with the median results for the A. ribosomal load, and B. proteins per mRNA molecule, for the 3'UTR linear (LIN) and non-linear (MARS) predictors (Additional file 1: Supplementary Methods). The best features according to the number of predictors they participated in (Additional file 1: Supplementary Methods) of the C. ribosomal load predictor, and D. proteins per mRNA molecule predictor, for the 3'UTR linear (LIN) and non-linear (MARS) predictors (Additional file 1: Supplementary Methods).

At the next stage, we aimed at understanding the quality of prediction that can be gained when using features of each of the main parts of the transcript (5'UTR, ORF, and 3'UTR) separately. Such an analysis can help us understand the relative contribution of each stage of translation (initiation, elongation, termination) to the overall translation efficiency. In addition, we aimed at better understanding the relevant gene expression features of each of these three parts as shaped by evolution. Furthermore, as there is much redundancy among the different features, such that certain features may mask other important ones in the combined regressor, we inferred the five aforementioned predictors (PA, RD, mRNA, RL and PPR) on the basis of the transcript's three main parts *separately*. A summary of the results appears in Figures 4, 5, 6, 7, 8, 9.

As can be seen, the correlations obtained for the linear and non-linear predictors respectively based on the features of the ORF alone (Figure 6A-C and 7A-B) are very similar to the ones obtained when considering the entire transcript (Figure 2A-C and 3A-B), correlations of 0.71/0.72 with PA (based on 20/12 features on average), 0.67/0.69 with RD (25/12 features on average), 0.67/0.68 with mRNA (20/11 features on average), 0.80/0.81 with RL (22/10 features on average), and 0.61/0.62 with PPR (19/9 features on average) respectively (all p-values <$10^{-35}$), suggesting that for inferring a good predictor of endogenous gene expression, the information in the UTRs is redundant. This result supports the conjecture that though some of the gene expression regulation mechanisms are known to be encoded mainly in the UTRs (*e.g*. mRNA degradation and translation initiation), evolution shaped ORFs in such a way that gene expression measurements can be inferred accurately based on the ORF alone.

The correlations of the linear and non-linear predictors respectively that are based on the UTRs were markedly lower: correlations of 0.27/0.21 with PA (8/3 features on average), 0.32/0.30 with RD (13/5 features on average), 0.27/0.26 with mRNA (10/4 features on average), 0.34/0.32 with RL (9/7 features on average), and 0.21/0.14 with PPR (3/5 features on average) respectively in the case of the 5'UTR features based predictors (all p-values <$10^{-5}$); correlations of 0.25/0.18 with PA (11/7 features on average), 0.36/0.34 with RD (18/4 features on average), 0.54/0.54 with mRNA (11/8 features on average), 0.61/0.61 with RL (13/5 features on average), and 0.52/0.47 with PPR (10/2 features on average) respectively in the case of the 3'UTR features based predictor (all p-values <$10^{-5}$).

The relevant features in the case of the 5'UTR (Figures 4, 5) in all the gene expression measurements include the folding strength (and GC content) at the end of the 5'UTR, which is related to translation initiation efficiency via ribosomal binding efficacy [8,9,48]. An additional feature is the length of the 5'UTR which is shorter for highly expressed genes (average length of 67.88 for the top 2% highly expressed genes, as opposed to 82.58 for the rest of the genes). Finally, many features are related to alternative translation initiation from the 5'UTR and include the number of alternative ATGs, their distance from the beginning of the ORF, and the optimality of the nucleotide context of the alternative ATGs to translation initiation [39,40]. These features may affect the rate and efficiency of translation initiation of the major ORF in the transcript and thus effect in a casual way the PA, RD, PPR, and RL; the mRNA is probably related to these variable in a non direct/causal way: highly expressed genes (e.g. in terms of PA and RD) are selected for efficient translation initiation and are also selected for higher mRNA levels.

The relevant features in the case of the ORF (Figures 6, 7) are generally similar to the ones obtained for the entire transcript (Figures 2, 3). Specifically, they include the tAI, features related to mRNA folding, and ORF length.

In addition, also in this case, the predictors included features related to the frequency of codons and amino acids; for example:

The frequency of the codons CCC, TTG, and GGT appear in the ORF based mRNA predictor and tend to have negative coefficients; the codons AGG and ATA tend to appear in the ORF based RD predictor with negative and positive coefficients respectively; the codons CGA and GCC tend to appear in the ORF based PA predictor with positive coefficients; the codons GCC and ACC tend to appears in the ORF based PPR predictor with positive coefficients; and the codons CCC and CGA tend to appear in the ORF based RL predictor with negative coefficients respectively. These results support the conjecture that the frequency of the different codons affect various aspects of gene expression in a way not modeled via conventional measures such as tAI, CAI, TASEP, etc.

Interestingly, the most relevant features in the case of the 3'UTR (Figures 8, 9) are similar to the ones obtained for the 5'UTR. The top features include the 3'UTR length and aspects of its mRNA folding. Additional features are related to possible alternative translation initiation from the 3'UTR, and include the number of alternative ATGs, their distance from the end of the ORF, and the optimality of the nucleotide context of the alternative ATGs to translation initiation. This is possibly related to the fact that during the eukaryotic translation initiation there is interaction between the 3' end (poly A at the 3'UTR) of the transcript and the initiation complex at the 5' end (5'UTR) of the transcript [6]; the pre-initiation complexes scanning the 5'end of the transcript may diffuse to its 3'end with high probability and perform undesired initiation event that are selected against in highly expressed genes.

### Predictors including features of the transcript optimized via mRNA levels

In this section, we briefly report the results obtained based on transcript features, of which some (*e.g*. tAI, ATG context, TASEP, etc), include parameters that were optimized/inferred based on mRNA levels measurements. For example, the tAI includes weights corresponding to codon-tRNA interaction efficiency which were inferred based on the correlation between the tAI and mRNA levels in *S. cerevisiae *[42].

First, it is not clear if such an optimization can improve the predictions. Second, it is interesting to see the predictions of variables such as PA and PPR based on the mRNA dependent features (see Additional file 1: Supplementary Methods for more details).

In the current analyses, the set of transcript features includes 5432 features (see Additional files 1, 2, 3 for a detailed description including list of features and default value of the features in each predictor). All the detailed results appear in the Additional file 1 (supplementary material); here we only report the highlights.

First, we investigated how well measures of gene expression can be predicted based on *all *the features of the transcript, when optimizing the relevant features via mRNA levels. Figure S1A-C in Additional file 1 includes the dot plot and correlation of the predicted *vs*. real protein levels (A), ribosomal densities (B), mRNA levels(C), and Figure S2A-B in Additional file 1 includes the dot plot and correlation of the predicted *vs*. estimated ribosomal load (A), and proteins per mRNA molecule (B), respectively, for the median linear predictors (Additional file 1: Supplementary Methods). As can be seen in Additional file 1: Figures S1-S2 all the correlations are significantly high -- a correlation of 0.77 with protein levels (based on 18 features on average), 0.67 with ribosomal density (based on 20 features on average), 0.92 with ribosomal load (based on 21 features on average), and 0.71 with proteins per mRNA molecule (based on 19 features on average), (all p-values < 10^-141^).

From the results we learn that the prediction of post-transcriptional aspects of gene expression (*i.e*. measurements that are not mRNA levels) cannot be improved significantly when adding mRNA levels information indirectly, *i.e*. features derived from it. In addition, the results demonstrate that combining the machine learning and biophysical approach can yield improved correlation with PA than the one obtained before for each of the approaches separately [3,7]. One central feature that appears in almost all the entire transcript based predictors (RD, PPR, mRNA, RL) is the Totally Asymmetric Exclusion Process (TASEP); as mentioned this feature is based on a biophysical simulation of gene translation and considers the adaptation of codons to the tRNA pool but also (among other aspects) the order of codons. Thus, this result supports the conjecture that the order of codons and not only their content/average value has important contribution to various gene expression aspects, and evolution shapes the codon order in endogenous genes to optimize the different stages of the gene expression process [36,40,50,54].

## Conclusions

We report a new strategy for predicting and analyzing gene expression that is based on exploiting features of the transcript, performing feature selection, and merging them via a regression model. The study connects features of the transcript shaped by its evolution and measurements of various steps of gene expression.

The results gained in this study are numerous and are founded on deep biophysical analyses and modeling of this process. Amongst others, we show that different stages of the gene expression process can be predicted with very high accuracy (all correlations above 0.61) based on only around 10-24 features (for the regressors based on the entire transcript), which are based solely on transcript nucleotide/codon composition. We show that PPR predictors based on ORF features are significantly more qualitative (twice higher correlation) than predictors based on the UTRs alone; this result supports the hypothesis that translation elongation (and not only initiation) is also a rate limiting stage of gene translation, and affects translation in a causal or non-causal way; thus, aspects of this process are encoded in the ORF, and evolution shapes ORFs' content based on the proteins they encode, but also based on their gene expression regulation.

It is important to understand that the causal relations reported in this study, based on endogenous genes, are not always clear; this is related to the fact that often highly expressed genes are under evolutionary selection for various features that ***do not ***improve translation in a direct way. Thus, these features may have significant correlations with genes' protein levels, which are not causal, nor do they affect their translation efficiency. For example, the frequency of an amino acid in a gene can have high correlation with its protein levels due to the specific functionality of the highly expressed proteins, and not due to the fact that this amino acid indeed improves the translation rate.

One interesting result reported here is the significant correlations between the transcript based predictors of mRNA levels and the actual mRNA levels (correlation of 0.68). This is surprising since it is assumed that transcription is mainly regulated via the promoter (which is not part of the transcript), while translation is regulated via patterns that appear in the transcript. This result supports the conjecture that aspects of the mRNA elongation and degradation steps (and not only translation) are also partially encoded in the transcript and thus may affect its evolution.

The reported results suggest several interesting future directions:

First, in this study we decided to concentrate on *S. cerevisiae *as this organism has the most high quality large scale measurements of gene expression. It will be interesting to generalize the reported results to other organisms, including multi-cellular organisms, when such data is available.

Second, as aforementioned, one interesting conclusion reported in this study is the predictors based on the ORF are significantly better than those based on the UTRs; and that the information encoded in the UTRs is redundant as it does not significantly improve the ORF prediction. It will be challenging to show that this conclusion is not due to the fact that the ORF simply tends to be longer than the UTRs (mean ORF length is 1490.8 while mean 5'UTR/3'UTR lengths are 82.33/133.62). This is not a trivial task as there is no simple mathematical model that describes the way regulatory information is encoded in the transcript considering the interaction of the transcript with other cellular properties, and the overlapping of different types of information encoded in it (*e.g*. the amino acid content of a protein; see the Additional file 1: Supplementary Methods regarding initial analyses we performed to answer this question).

Third, the features inferred here can teach us about transcript evolution and the way its expression aspect constraints its evolution. We show that various expression aspects of genes can be predicted solely based on their transcript; specifically, that highly expressed genes tend to have specific codons, shorter UTRs, improved tAI and CAI, weak/strong folding in different parts of the transcript, and more. These features probably tend to optimize expression in various ways; thus, the results reported here support the conjecture that 'synonymous' mutations (in terms of the effect on the amino acid content) influencing these features should affect the fitness of the organism, and thus should not be treated as synonymous. Various such mutations have been previously reported [5,55]; the long list of features reported here may provide additional such cases, which can be considered when estimating non-neutral/neutral evolution [56,57]. More generally, the results reported in the current study suggest that the ORF and UTRs of a gene are shaped by the different stages of their expression levels, thus we need to consider gene expression when developing models for studying genome evolution.

Finally, we believe that the lists of new relevant features reported in the current study, which are based on the way evolution shapes the expression of endogenous genes, can teach us about novel mechanisms related to gene expression regulation and modeling. Specifically, we report a set of codons and codon pairs that have significant effect on the prediction quality given traditional measures of codon bias and elongation efficiency. These features may affect expression levels via various mechanisms including: 1) regulation of tRNA levels not accurately modeled in current codon bias and translation elongation features/indexes [32,37,42]; 2) translation frame shifts [58]; 3) transcription elongation efficiency [59]; 4) and tRNA recycling [54]. To better understand the biophysical rules of these features and to infer causality, we suggest to explore them via experiments that include introducing them into a reporter gene and measuring the effect of these features on changes in its expression levels measurements, and/or by multi-organism studies of their evolutionary patterns.

## Competing interests

The authors declare that they have no competing interests.

## Authors' contributions

HZ and TT conceived the research, analyzed the data, and wrote the paper.

## Supplementary Material

Additional file 1 **Contains the supplementary methods and some additional results**. Click here for file

Additional file 2 **Main scheme regression features**. A short description of all the features utilized in the study, in the main regressor scheme. Click here for file

Additional file 3 **Expression dependant scheme regression features**. A short description of all the features utilized in the expression dependant scheme. Click here for file

## Declarations

Publication of this article was funded by Tel-Aviv University.

This article has been published as part of BMC Bioinformatics Volume 14 Supplement 15, 2013: Proceedings from the Eleventh Annual Research in Computational Molecular Biology (RECOMB) Satellite Workshop on Comparative Genomics. The full contents of the supplement are available online at http://www.biomedcentral.com/bmcbioinformatics/supplements/14/S15.

## References

[B1] Vogel C de Sousa Abreu R Ko D Le SY Shapiro BA Burns SC Sandhu D Boutz DR Marcotte EM Penalva LO Sequence signatures and mRNA concentration can explain two-thirds of protein abundance variation in a human cell line Molecular systems biology 2010 14 1 10.1038/msb.2010.59 PMC2947365 20739923

[B2] Drummond DA Wilke CO The evolutionary consequences of erroneous protein synthesis Nature Reviews Genetics 2009 14 10 715 724 10.1038/nrg2662 19763154 PMC2764353

[B3] Tuller T Kupiec M Ruppin E Determinants of protein abundance and translation efficiency in S. cerevisiae PLoS computational biology 2007 14 12 e248 10.1371/journal.pcbi.0030248 18159940 PMC2230678

[B4] Gingold H Pilpel Y Determinants of translation efficiency and accuracy Molecular systems biology 2011 14 1 10.1038/msb.2011.14 PMC3101949 21487400

[B5] Plotkin JB Kudla G Synonymous but not the same: the causes and consequences of codon bias Nat Rev Genet 2010 14 1 32 42 21102527 10.1038/nrg2899 PMC3074964

[B6] Alberts B Johnson A Lewis J Raff M Roberts K Walter P Molecular Biology of the Cell (Garland Science, New York, 2002) b) M Bogyo, M Gaczynska, HL Ploegh, Biopolymers 2002 14 269 280

[B7] Reuveni S Meilijson I Kupiec M Ruppin E Tuller T Genome-scale analysis of translation elongation with a ribosome flow model PLoS computational biology 2011 14 9 e1002127 10.1371/journal.pcbi.1002127 21909250 PMC3164701

[B8] Tuller T Waldman YY Kupiec M Ruppin E Translation efficiency is determined by both codon bias and folding energy Proc Natl Acad Sci USA 2010 14 8 3645 3650 10.1073/pnas.0909910107 20133581 PMC2840511

[B9] Kudla G Murray AW Tollervey D Plotkin JB Coding-sequence determinants of gene expression in Escherichia coli Science 2009 14 5924 255 258 10.1126/science.1170160 19359587 PMC3902468

[B10] Coghlan A Wolfe KH Relationship of codon bias to mRNA concentration and protein length in Saccharomyces cerevisiae Yeast 2000 14 12 1131 1145 10.1002/1097-0061(20000915)16:12<1131::AID-YEA609>3.0.CO;2-F 10953085

[B11] Lercher MJ Urrutia AO Pavlíč A Hurst LD A unification of mosaic structures in the human genome Human molecular genetics 2003 14 19 2411 2415 10.1093/hmg/ddg251 12915446

[B12] Pál C Papp B Hurst LD Highly expressed genes in yeast evolve slowly Genetics 2001 14 2 927 931 11430355 10.1093/genetics/158.2.927 PMC1461684

[B13] Krylov DM Wolf YI Rogozin IB Koonin EV Gene loss, protein sequence divergence, gene dispensability, expression level, and interactivity are correlated in eukaryotic evolution Genome Research 2003 14 10 2229 2235 10.1101/gr.1589103 14525925 PMC403683

[B14] Churchill GA Fundamentals of experimental design for cDNA microarrays Nature genetics 2002 14 supp 490 495 10.1038/ng1031 12454643

[B15] Ghaemmaghami S Huh WK Bower K Howson RW Belle A Dephoure N O'Shea EK Weissman JS Global analysis of protein expression in yeast Nature 2003 14 6959 737 741 10.1038/nature02046 14562106

[B16] Newman JRS Ghaemmaghami S Ihmels J Breslow DK Noble M DeRisi JL Weissman JS Single-cell proteomic analysis of S. cerevisiae reveals the architecture of biological noise Nature 2006 14 7095 840 846 10.1038/nature04785 16699522

[B17] Lu P Vogel C Wang R Yao X Marcotte EM Absolute protein expression profiling estimates the relative contributions of transcriptional and translational regulation Nature biotechnology 2006 14 1 117 124 17187058 10.1038/nbt1270

[B18] Arava Y Wang Y Storey JD Liu CL Brown PO Herschlag D Genome-wide analysis of mRNA translation profiles in Saccharomycescerevisiae Proceedings of the National Academy of Sciences of the United States of America 2003 14 7 3889 10.1073/pnas.0635171100 12660367 PMC153018

[B19] Ingolia NT Ghaemmaghami S Newman JRS Weissman JS Genome-wide analysis in vivo of translation with nucleotide resolution using ribosome profiling Science 2009 14 5924 218 10.1126/science.1168978 19213877 PMC2746483

[B20] Taniguchi Y Choi PJ Li GW Chen H Babu M Hearn J Emili A Xie XS Quantifying E. coli proteome and transcriptome with single-molecule sensitivity in single cellsscience 2010 14 5991 533 538 10.1126/science.1188308 20671182 PMC2922915

[B21] Baerenfaller K Grossmann J Grobei MA Hull R Hirsch-Hoffmann M Yalovsky S Zimmermann P Grossniklaus U Gruissem W Baginsky S Genome-scale proteomics reveals Arabidopsis thaliana gene models and proteome dynamicsscience 2008 14 5878 938 941 10.1126/science.1157956 18436743

[B22] Wen JD Lancaster L Hodges C Zeri AC Yoshimura SH Noller HF Bustamante C Tinoco I Following translation by single ribosomes one codon at a time Nature 2008 14 7187 598 603 10.1038/nature06716 18327250 PMC2556548

[B23] Uemura S Aitken CEÃ Korlach J Flusberg BA Turner SW Puglisi JD Real-time tRNA transit on single translating ribosomes at codon resolution Nature 2010 14 7291 1012 1017 10.1038/nature08925 20393556 PMC4466108

[B24] Kertesz M Wan Y Mazor E Rinn JL Nutter RC Chang HY Segal E Genome-wide measurement of RNA secondary structure in yeast Nature 2010 14 7311 103 107 10.1038/nature09322 20811459 PMC3847670

[B25] Guo H Ingolia NT Weissman JS Bartel DP Mammalian microRNAs predominantly act to decrease target mRNA levels Nature 2010 14 7308 835 840 10.1038/nature09267 20703300 PMC2990499

[B26] Lackner DH Beilharz TH Marguerat S Mata J Watt S Schubert F Preiss T Bähler J A network of multiple regulatory layers shapes gene expression in fission yeast Molecular cell 2007 14 1 145 155 10.1016/j.molcel.2007.03.002 17434133 PMC1885965

[B27] Shalem O Dahan O Levo M Martinez MR Furman I Segal E Pilpel Y Transient transcriptional responses to stress are generated by opposing effects of mRNA production and degradation Molecular systems biology 2008 14 1 10.1038/msb.2008.59 PMC2583085 18854817

[B28] Narsai R Howell KA Millar AH O'Toole N Small I Whelan J Genome-wide analysis of mRNA decay rates and their determinants in Arabidopsis thaliana The Plant Cell Online 2007 14 11 3418 3436 10.1105/tpc.107.055046 PMC2174890 18024567

[B29] Futcher B Latter G Monardo P McLaughlin C Garrels J A sampling of the yeast proteome Molecular and Cellular Biology 1999 14 11 7357 7368 10523624 10.1128/mcb.19.11.7357 PMC84729

[B30] Dittmar KA Goodenbour JM Pan T Tissue-specific differences in human transfer RNA expression PLoS genetics 2006 14 12 e221 10.1371/journal.pgen.0020221 17194224 PMC1713254

[B31] Dittmar KA Sørensen MA Elf J Ehrenberg M Pan T Selective charging of tRNA isoacceptors induced by amino-acid starvation EMBO reports 2005 14 2 151 157 10.1038/sj.embor.7400341 15678157 PMC1299251

[B32] Sharp PM Li WH The codon adaptation index-a measure of directional synonymous codon usage bias, and its potential applications Nucleic acids research 1987 14 3 1281 1295 10.1093/nar/15.3.1281 3547335 PMC340524

[B33] Huang T Wan S Xu Z Zheng Y Feng KY Li HP Kong X Cai YD Analysis and prediction of translation rate based on sequence and functional features of the mRNA PLos one 2011 14 1 e16036 10.1371/journal.pone.0016036 21253596 PMC3017080

[B34] MacDonald CT Gibbs JH Pipkin AC Kinetics of biopolymerization on nucleic acid templates Biopolymers 1968 14 1 1 25 10.1002/bip.1968.360060102 5641411

[B35] Heinrich R Rapoport TA Mathematical modelling of translation of mRNA in eucaryotes; steady states, time-dependent processes and application to reticulocytest Journal of Theoretical Biology 1980 14 2 279 313 10.1016/0022-5193(80)90008-9 7442295

[B36] Tuller T Carmi A Vestsigian K Navon S Dorfan Y Zaborske J Pan T Dahan O Furman I Pilpel Y An evolutionarily conserved mechanism for controlling the efficiency of protein translation Cell 2010 14 2 344 354 10.1016/j.cell.2010.03.031 20403328

[B37] Shaw LB Zia R Lee KH Totally asymmetric exclusion process with extended objects: A model for protein synthesis Physical Review E 2003 14 2 021910 10.1103/PhysRevE.68.021910 14525009

[B38] Zhang S Goldman E Zubay G Clustering of low usage codons and ribosome movement Journal of Theoretical Biology 1994 14 4 339 354 10.1006/jtbi.1994.1196 7996861

[B39] Kozak M Point mutations define a sequence flanking the AUG initiator codon that modulates translation by eukaryotic ribosomes Cell 1986 14 2 283 292 10.1016/0092-8674(86)90762-2 3943125

[B40] Zur H Tuller T New Universal Rules of Eukaryotic Translation Initiation Fidelity PLoS Comput Biol 2013 in press 10.1371/journal.pcbi.1003136 PMC3708879 23874179

[B41] Ben-Yehezkel* T Zur* H Marx T Shpiro E Tuller T Mapping the Translation Initiation Landscape of an *S. cerevisiae *Gene Using Fluorescent Proteins Genomics 2013 in press 10.1016/j.ygeno.2013.05.003 23726901

[B42] dos Reis M Savva R Wernisch L Solving the riddle of codon usage preferences: a test for translational selection Nucleic Acids Res 2004 14 17 5036 5044 10.1093/nar/gkh834 15448185 PMC521650

[B43] Tuller T Veksler-Lublinsky I Gazit N Kupiec M Ruppin E Ziv-Ukelson M Composite effects of gene determinants on the translation speed and density of ribosomes Genome biology 2011 14 11 R110 10.1186/gb-2011-12-11-r110 22050731 PMC3334596

[B44] Breiman L Random forests Machine learning 2001 14 1 5 32 10.1023/A:1010933404324

[B45] Friedman JH Multivariate adaptive regression splines The annals of statistics 1991 1 67

[B46] Qian W Yang J-R Pearson NM Maclean C Zhang J Balanced codon usage optimizes eukaryotic translational efficiency PLoS genetics 2012 14 3 e1002603 10.1371/journal.pgen.1002603 22479199 PMC3315465

[B47] Tuller T Waldman YY Kupiec M Ruppin E Translation efficiency is determined by both codon bias and folding energy Proceedings of the National Academy of Sciences 2010 14 8 3645 3650 10.1073/pnas.0909910107 PMC2840511 20133581

[B48] Gu W Zhou T Wilke CO A universal trend of reduced mRNA stability near the translation-initiation site in prokaryotes and eukaryotes PLoS Comput Biol 2010 2010 14 2 1 8 10.1371/journal.pcbi.1000664 PMC2816680 20140241

[B49] Zur H Tuller T Strong association between mRNA folding strength and protein abundance in S. cerevisiae EMBO Rep 2012 10.1038/embor.2011.262 PMC3323128 22249164

[B50] Tuller T Veksler-Lublinsky I Gazit N Kupiec M Ruppin E Ziv-Ukelson M Composite Effects of Gene Determinants on the Translation Speed and Density of Ribosomes Genome Biol 2011 14 11 R110 10.1186/gb-2011-12-11-r110 22050731 PMC3334596

[B51] Duret L Mouchiroud D Expression pattern and, surprisingly, gene length shape codon usage in Caenorhabditis, Drosophila, and Arabidopsis Proceedings of the National Academy of Sciences 1999 14 8 4482 4487 10.1073/pnas.96.8.4482 PMC16358 10200288

[B52] Shalgi R Lapidot M Shamir R Pilpel Y A catalog of stability-associated sequence elements in 3'UTRs of yeast mRNAs Genome biology 2005 14 10 10.1186/gb-2005-6-10-r86 PMC1257469 16207357

[B53] Vreken P van der Veen R de Regt V de Maat A Planta R Raue H Turnover rate of yeast PGK mRNA can be changed by specific alterations in its trailer structure Biochimie 1991 14 6 729 737 10.1016/0300-9084(91)90053-4 1764519

[B54] Cannarozzi G Schraudolph NN Faty M Von Rohr P Friberg MT Roth AC Gonnet P Gonnet G Barral Y A role for codon order in translation dynamics Cell 2010 14 2 355 367 10.1016/j.cell.2010.02.036 20403329

[B55] Chamary JV Parmley JL Hurst LD Hearing silence: non-neutral evolution at synonymous sites in mammals Nat Rev Genet 2006 14 2 98 108 10.1038/nrg1770 16418745

[B56] Li W-H Wu C-I Luo C-C A new method for estimating synonymous and nonsynonymous rates of nucleotide substitution considering the relative likelihood of nucleotide and codon changes Molecular Biology and Evolution 1985 14 2 150 174 3916709 10.1093/oxfordjournals.molbev.a040343

[B57] Nei M Gojobori T Simple methods for estimating the numbers of synonymous and nonsynonymous nucleotide substitutions Molecular Biology and Evolution 1986 14 5 418 426 3444411 10.1093/oxfordjournals.molbev.a040410

[B58] Schwartz R Curran JF Analyses of frameshifting at UUU-pyrimidine sites Nucleic acids research 1997 14 10 2005 2011 10.1093/nar/25.10.2005 9115369 PMC146683

[B59] Churchman LS Weissman JS Nascent transcript sequencing visualizes transcription at nucleotide resolution Nature 2011 14 7330 368 373 10.1038/nature09652 21248844 PMC3880149

