# Supplementary material for: Transcript features alone enable accurate prediction and understanding of gene expression in S. cerevisiae
Source: BMC Bioinformatics. 2013 Oct 15;14(Suppl 15):S1. doi: 10.1186/1471-2105-14-S15-S1 (PMC3852043; doi:10.1186/1471-2105-14-S15-S1)
Supplement: Additional file 1 — Contains the supplementary methods and some additional results. [file 1471-2105-14-S15-S1-S1.PDF]

Supplementary of ‘Transcript Features Alone Enable Accurate Prediction and Understanding of Gene Expression in *S. cerevisiae*’

Hadas Zur[1] and Tamir Tuller[2§]

[1]Blavatnik School of Computer Science, Tel Aviv University, Israel.

[2]Department of Biomedical Engineering, the Engineering Faculty, Tel Aviv University, Israel.

TT: tamirtul@post.tau.ac.il

Supplementary Methods

Method Summary

First large scale genomic and expression data, including mRNA levels, proteins per mRNA molecule (PPR), ribosomal load (RL), ribosomal densities, ORF sequences and UTRs, for thousands of S. cerevisiae endogenous genes were gathered from various sources, and were normalized to remove biases and batch effects (see sub-section ‘Data Sources’ for more details).

At the next step, we generated a large set of features (5323 features) that are related to various properties of the transcript, biological knowledge of aspects of gene expression, and properties of the intracellular environment of S. cerevisiae. Computing many of these features required implementing various modeling, computational and statistical approaches, optimizations, and predictions of biophysical interactions and properties. These features include among others, nucleotide and amino acid compositions in various parts of the transcripts, computational prediction of the strength of the RNA folding of different parts of the transcript, adaptations of codons to the cellular tRNA pool, models of translation

initiation, models of translation elongation, and more (see sub-section 'Transcript sequence features').

Next, in order to design accurate predictors of the aforementioned gene expression measures, based only on features of its transcripts, we devised the following regression scheme:

We built linear and non linear predictors based on the features mentioned above for the genes' sequence segments, the 5'UTR, ORF and 3'UTR separately, and also combining the three together, in the following manner. The data was divided into terciles: a train, test and validation set, performing this sampling 100 times, thus resulting with 100 predictors per segment/entire transcript. This was done in-order to avoid overfitting, and show that there does not exist a single predictor encapsulating the information in each region of the transcript in its entirety, but much like the nucleotide context of a potential initiating ATG, a statistical distribution of features influencing each stage of gene expression. Additionally, this enabled us to perform statistical analyses of the prevalence, and thus significance of features. We implemented a greedy feature selection process, by which in each iteration every feature is added respectively to the growing regressor, and the feature contributing to the highest correlation is selected, starting with a vector of ones for the linear regressor, and an empty model for the non-linear regressor. At the end of each stage, the current predicted regressor coefficients of the selected features are assessed on the test set. The selected regressor is then evaluated on the validation set, in order to avoid overfitting. We used the Spearman rank correlation coefficient for detecting monotonic trends, because the Pearson product moment correlation coefficient is strongly biased towards linear trends. Thus, all features were ranked prior to regression analysis.

To model non-linearity we used Multivariate adaptive regression splines (MARS), which is a form of regression analysis introduced in [1]. It is a non-parametric regression technique, and can be seen as an extension of linear models that automatically models non-linearities and interactions. MARS approximates non-linear relationships with continuous piecewise linear functions (see sub-section 'Transcript sequence predictors' for more details).

Data sources

In the current paper our aim is to study transcript features related to typical/average stages of gene expression. To this end, we averaged (when possible) the measures of expression levels (*e.g.* mRNA, PA, PPR) from various sources in standards growth conditions (YPD) after normalizing each of the sources such that all of them will have an identical average, as described in detail below.

Thus, our analyses are not sensitive to condition specific changes in gene expression. However, the research question of the current study is very interesting and relevant. The main explanation for the relevance of our research question is the fact that there is high correlation

between the expression levels of genes in various conditions (i.e. genes that tend to be highly expressed in one condition tend to be highly expressed also in different conditions; they may change their expression in different conditions but their relative ranking remains similar); for example, highly used measures such as the codon adaptation index (CAI; [2]) and the tRNA adaptation index (tAI; [3]) are based on such assumption.

Indeed, for example, previous influential papers in the field have studied questions related to evolutionary rates of genes and their expression levels using gene expression definitions similar to ours [4, 5]

UTRs and coding sequences: The *S. cerevisiae* genome was downloaded from the Biomart Ensembl database, for 5861 genes. *S. cerevisiae* 5'UTRs and 3'UTRs were obtained from [6]. The lengths and composition of 5'UTRs and 3'UTRs are known for 4367 and 4972 genes respectively, for the 5861 *S. cerevisiae* genes we obtained. The mean length of known 5'UTRs is 82.33, while for 3'UTRs it is 133.62, with the mean ORF lengths being 1490.8. In this study we only utilized genes with known 5'UTRs, thus 4367 genes participated (for which the 3'UTRs are known).

Ribosomal densities: For *S. cerevisiae* we consider two large scale Ribosomal Density (RD) measurements (the number of ribosomes occupying the transcript divided by its length); each generated by a different technology. The first dataset was generated more recently by Ingolia *et al*. [7] (4648 genes, and included values for 3954 /4367 genes participating in the study), and the second by [8] (5181 genes, and included values for 4015/4367 genes participating in the study). We average across the two RD datasets (after normalizing each dataset by its mean), in order to minimize experimental noise (resulting with 4316 genes, which included values for 3682/4367 genes participating in the study). Similar results were obtained when we analyzed each dataset separately.

mRNA levels: For *S. cerevisiae* we considered the large scale measurements of mRNA levels from [7]. There are measurements for 5295 genes, and included values for 4176 /4367 genes participating in the study.

Protein Abundance : For *S. cerevisiae* we considered four quantitative large scale measurements of Protein Abundance (PA): [9] (3839 genes, and included values for 3263/4367 genes participating in the study), two large scale measurements in two conditions from [10] (2508/2433 genes, and included values for 2250 and 2187/4367 genes participating in the study respectively), and large scale protein abundance from [11] (2360 genes, and included values for 2117/4367 genes participating in the study). Similarly to the RD, we averaged across the four datasets to reduce experimental noise (resulting with 1448 genes, which included values for 1343/4367 genes participating in the study). Similar results were obtained when we analyzed each dataset separately.

Metabolic costs of amino acids: Data for metabolic cost of amino acids for *S. cerevisiae* came from [12]. These are utilized in the regressor version including features optimized via expression levels.

Proteins per mRNA molecule (PPR), (Protein Abundance)/(mRNA levels): is the number of proteins produced on average from an mRNA molecule, we termed this proteins per mRNA molecule, or PPR for short. We added this feature as it is directly related to the translation stage, unlike PA which is related to both transcription and translation. The final number of proteins (PA) is related to mRNA levels the output of the transcription stage, and the post-transcriptional regulatory stages related to generating proteins from the mRNA sequence (e.g. gene translation and protein degradation).

Ribosomal-Load, (mRNA levels)· (ribosomal density) (RL): The number of ribosomes consumed by a gene is proportional to its mRNA levels and the ribosomal density on the mRNA molecules transcripted from the gene. Thus, we used (mRNA levels)·(ribosomal density), which we named Ribosomal-Load (RL), as a measure of the number of translation events of a gene.

Features

Transcript sequence features

GC Content is the percentage of nitrogenous bases on a DNA molecule that are either guanine or cytosine. GC pairs are bound by three hydrogen bonds, while AT pairs are bound by two hydrogen bonds, thus DNA with high GC-content is more stable than DNA with low GC-content. The 5’UTR predictor contains 2 features, one representing the GC content across the entire 5’UTR, and the other of the last 40nt of the 5’UTR. Similarly the 3’UTR predictor contains a feature of GC content across the entire 3’UTR, and for the first 40nt. In accordance the ORF predictor contains the GC content across the entire coding sequence, but also for the first one hundred 40nt long windows with a slide of one nt. The combined predictor includes all the aforementioned features in addition to the GC content in the last 40nt of the 5'UTR and first 40nt of the ORF, entire 5'UTR and first 40nt of the ORF, entire 5'UTR and entire ORF, last 40nt of the ORF and first 40nt of the 3'UTR, last 40nt of the ORF and entire 3'UTR, and entire ORF and entire 3'UTR.

Predicted Folding Energy is an approximation of the mRNA secondary structure. We applied the Matlab rnafold function (Matlab Bioinformatics Toolbox), which predicts the folding energy of the secondary structure associated with the minimum free energy for an RNA sequence (or subsequence). To obtain the estimation of the mean local folding of a genomic sequence, we estimated the folding energy of all the sliding windows of length 40nt (slide of 1nt; 40nt is the approximated length of the ribosomes’ footprint [7]), and averaged the resultant folding energy prediction of all the windows induced by the sequence. Specifically, mean predicted local folding energies used in this study were performed based on the entire transcript of each gene (*i.e.* including 5’UTR, ORF, and 3'UTR) and for each

region (5’UTR, ORF, and 3'UTR) separately. Until recently, it was not feasible to perform actual measurements of folding energy, thus these approximations, which work relatively accurately only on short segments, were employed. We thought it would be interesting to compare the performance of the newly developed measured folding energy (PARS) [13], to the predicted one (this could help infer biases in the new technology). In addition since the PARS is a global feature, we could not utilize if for the individual transcript segments predictors, namely the 5’UTR/ORF/3’UTR, and it is only utilized in the regressor version including features optimized via expression levels. The same feature definitions as for GC content were used for the mean predicted folding energy.

Measured Folding Energy: Recently, a new technology for measuring folding strength of RNA sequences at single nucleotide resolution was developed [13]. The product of this method, named the Parallel Analysis of RNA Structure (PARS) score, includes the estimated ratio between the probability that each nucleotide in the transcript is in a double-stranded conformation and the probability that it is in a single-stranded conformation. The PARS score was computed in vitro for transcripts devoid of any ribosomes. As mRNA folding is a major feature of a transcript it may affect its translation rate, or may be related to its protein abundance in a non-causal way (*e.g.* via its relation to the mRNA levels). In a previous study, we utilized the availability of such a new tool, which has only been applied to *S. cerevisiae*, to analyze the relationship between mRNA folding strength and PA. As aforementioned we found that genes with high protein abundance tend to have strong mRNA folding, and high ribosomal density and mRNA levels, suggesting that this relation at least partially pertains to the efficiency of translation elongation [14], and showed that this relation is causal. The PARS is a global feature and thus relevant only to the combined predictor, and includes the mean PARS over each respective segment and over the entire transcript, first 40nt of the ORF, last 40nt of the 5'UTR, last 40nt of the 5'UTR and first 40nt of the ORF, entire 5'UTR and first 40nt of the ORF, entire 5'UTR and entire ORF, first 40nt of the 3'UTR, first 40nt of the ORF and first 40nt of the 3'UTR, first 40nt of the ORF and entire 3'UTR, entire ORF and entire 3'UTR. Additionally we took the exponent of each of the above mentioned features, as the PARS is a log based measure. In our previous study [14] we found that the exponent of the PARS achieved better correlations with the translation variables, such as protein abundance, ribosomal density, etc. The PARS features are utilized only in the regressor version including features optimized via expression levels.

ATG Context Score: Gene translation consists of three stages: initiation (the binding of the ribosome to the transcript and the association of the small and large subunits), elongation (the iterative translation of triplets of nucleotides to amino acids by the ribosome) and termination (the disassociation of the large and small subunits of the ribosome and the completion of the process), which form a recurring cycle of events. In eukaryotes the initiation step involves formation of a pre-initiation complex (consisting of the small subunit, 43S or the 40S subunit, and initiation tRNA). This complex accompanied by additional initiation factors scan the mRNA sequence starting from its 5' end towards its 3' end, until it finds a start codon (usually an AUG that is recognized by the initiation tRNA), which represents the beginning of the open reading frame (ORF). The recognition of the start codon triggers the association

of the large subunit and the beginning of the elongation step [15-18]. However, ATG codons are expected to be present in all possible reading frames, upstream and downstream of the main START ATG; how thus does the scanning pre-initiation complex recognize the start ATG? Over 30 years ago Kozak suggested that a specific context (*i.e.* the nucleotides before and after a codon) surrounding the initiating ATG codon is required for its recognition by the pre-initiation complex; asserting that this context should appear only in the vicinity of the initiating (START) ATG codon of the ORF [17]. In a previous study [19], we analyzed dozens of eukaryotic genomes demonstrating that there is rather weak selection for a specific ATG context near the START ATG of the ORF. In addition, we found that in all frame-shifts (there are three possible reading frames: frame 0 is identical to the reading frame of the gene ORF; frames 1 and 2 represent a frame shift of 1 or 2 nucleotides relative to the main frame) there is a universal trend of selection for low numbers of ATG codons in the proximity of the main START ATG. We further suggest that there is selection for *anti optimal* ATG contexts in the vicinity of the START ATG. Thus, the efficiency and fidelity of translation initiation is encoded in the 5'UTR but also at the beginning of the ORF. The observed nucleotide patterns suggest that in all the analyzed organisms the pre-initiation complex often misses the START ATG of the ORF, and may commence translation from an alternative, potentially frame-shifted, initiation start-site. Thus, to prevent the translation of undesired proteins, there is selection for nucleotide sequences with low affinity to the pre-initiation complex near the beginning of the ORF. For that purpose we devised a statistical score for ATG codons (named the ATG Context Score) [19] based on their similarity to the sequence context of main ATG START codons of highly expressed genes, in which higher context scores denote similarity to the ATG context of highly expressed genes. We calculate the context score according to the following algorithm (scheme):

1. Select percentage of highly expressed/translated genes. This mode is used in the regressor version including features optimized via expression levels. In our main regressor scheme which is independent of expression levels, the train set was utilized, making the context Score measure independent of expression levels. Results are robust to versions of this procedure.
2. Calculate a position specific scoring matrix (PSSM) based on the nucleotide context around the start codon of the selected highly-expressed/train-set genes. This PSSM represents the nucleotides necessary for highly efficient translation in the case of the highly-expressed genes.
3. Calculate the context score per ATG position according to the PSSM: $ATGcs_j = \exp\left(\sum_i log(P_{ij})\right)$, where j is the gene index, i the nucleotide position, P_{ij} the probability that the i*th* nucleotide of the j*th* gene appears in the i*th* position. The *relative* context score is calculated by normalizing the gene's ATG context score by that of the gene's main START ATG: $ATGcs_j = \exp\left(\sum_i log(P_{ij})\right)/\exp\left(startATGcs_j\right)$, where $startATGcs_j$ is the context score of the first ATG in the ORF (main START codon) of the j*th* gene.

We did not consider genes without reconstructed 5'UTRs or with 5'UTRs shorter than 6nt. The following subsections include more details regarding stages 1-2.

1. Training set selection

The training set for the PSSM calculation is based on the selection of the top 2% of highly expressed genes, according to the product of ribosomal density (RD) and mRNA levels (ML) [7, 8], which represents the total flux of ribosomes over a gene. However, similar results were achieved when selecting the highly expressed group of genes based solely on RD or ML, and also when based on other measures of expression, such as protein abundance etc. In order to avoid over fitting, we select the top 4% of the highly expressed genes and then randomly select half of that group as the training set. Different percentages of training set sizes (up to 50%) were also tested in the same manner, and the results are robust to training set size variance. This mode is used in the regressor version including features optimized via expression levels. In our main regressor scheme which is independent of expression levels, the train set was utilized, making the context Score measure independent of expression levels.

2. PSSM Calculation

The PSSM was calculated according to the nucleotide (C, A, T, G) appearance probability (frequency), of the training set (2% highly expressed genes selected at (1), or regressor train set accordingly), for 6 nts before and 3 nts after the first ATG (start) codon, based on the length of the potential optimal ATG context proposed by Hamilton *et al.* [20]. We achieved similar results when considering various context lengths. Interval values between 3 to 48nts around the start codon were examined, for symmetric and asymmetric combinations, in jumps of 3nts, and additionally in jumps of 1nt from 1nt up to 10nts, and the results are robust to interval variance. These intervals were tested for all the aforementioned training set sizes in (1), and the results remain robust.

3. ATG Context Score Profile Calculation

Utilizing the context score measure, we calculated a position specific ATG context score profile spanning the position specific ATG appearance in the entire transcript, where for each gene, for each in-frame/frame-shifted ATG with the main START codon, a context score was calculated. Training set genes were excluded at this stage to avoid over-fitting. Looking at the mean and maximum ATG context scores and relative context scores, across the transcript segments (5’UTR/ORF/3’UTR) separately and all together, for all frames separately and combined, we included the following features in each respective predictor: across the entire and last 30 codons of the 5'UTR, across entire first 200 codons and first 30 codons of the ORF, across only the entire 3'UTR as for the first 30 codons there were too few features. The combined predictor included all the aforementioned features in addition to the last 30 codons of the 5'UTR and the first 30 codons of the ORF, last 30 codons of the ORF and first 30 codons of the 3'UTR.

ATG context score of the main START ATG of the ORF: this feature is relevant only to the ORF and combined predictor.

Number of ATGs features are defined for the same regions as those of the context score features.

Distance of the first ATG in the respective transcript segment from the main START ATG of the ORF, for all frames separately and together.

The number, mean and maximum length and metabolic cost of uORFs in the UTRs: An Upstream Open Reading Frame (uORF) is a very short Open Reading Frame (ORF) within the UTR. The 5'UTR predictor includes the number, mean and maximum length and metabolic cost of uORFs across the entire and last 30 codons of the 5'UTR. The 3'UTR predictor includes the number, mean and maximum length and metabolic cost of uORFs across the entire and first 30 codons of the 3'UTR. For both, the features are calculated for all frames separately and together. The combined predictor includes in addition to the aforementioned features, the number, mean and maximum length and metabolic cost of uORFs across the entire and last 30 codons of the 5'UTR allowing ending in the ORF. The total energy cost of amino acids in *S. cerevisiae* under respiratory conditions were taken from [12], and the metabolic cost of a peptide was calculated as the sum of the energy cost of the amino acids composing it. Peptide length was measured as the number of nucleotides composing it. The metabolic cost of peptides features were utilized only in the regressor version including features optimized via expression levels, as the computation of the amino acid metabolic costs relies on them to some extent.

The number mean and maximum length and metabolic cost of shifted ORFs (sORFs) in the coding sequence: An sORF (as we termed it) is a shifted truncated ORF, starting with an alternative ATG in the ORF and terminating with a stop codon. The ORF predictor includes the number, mean and maximum length and metabolic cost of sORFs across the entire first 200 codons and first 30 codons of the ORF. The combined regressor includes in addition to the above mentioned, the number, mean and maximum length and metabolic cost of sORFs across the entire and first 200 codons of the ORF allowing ending in the 3'UTR. For both, the features are calculated for all frames separately and together. The metabolic cost of peptides features were utilized only in the regressor version including features optimized via expression levels.

The number of Base Pairs: A base pair (bp) is the linking between two nitrogenous bases on RNA strands that are connected via hydrogen bonds. In the canonical Watson-Crick DNA base pairing, adenine (A) forms a base pair with uracil (U), and guanine (G) forms a base pair with cytosine (C). The numbers of base pairs were calculated based on the 5'UTR, ORF, 3'UTR and entire transcript bracket notation , which are based on real [13] or predicted measurements of *S. cerevisiae* mRNA folding, for the regressor scheme including features based on expression levels and the main one which does not, respectively. Each of the predictors includes the number of base pairs in its respective segment (5'UTR/ORF/3'UTR), with the combined predictor including in addition to those the number of base pairs across the entire sequence.

The Codon Adaptation Index (CAI) is a technique for analyzing codon usage bias. The CAI [2] measures the deviation of a given protein coding gene sequence with respect to a reference set of genes. Ideally, the reference set in CAI is composed of highly expressed genes, so that CAI provides an indication of gene expression levels under the assumption that there is translational selection to optimize gene sequences according to their expression levels. The rationale for this is dual: highly expressed genes need to compete for resources (*i.e.* ribosomes) in fast-growing organisms, and it makes sense for them to be also more accurately translated. Both hypotheses lead to highly expressed genes using mostly codons for tRNA species that are abundant in the cell. The CAI is simply defined as the geometric mean of the weight associated to each codon over the length of the gene sequence (measured in codons):

$$CAI = \exp\,(1/L\sum_{l=1}^{L}\ln\,(w_i(l)))$$

For each amino acid, the weight of each of its codons, in CAI, is computed as the ratio between the observed frequency of the codon (f_i) and the frequency of the synonymous codon (f_j) for that amino acid:

$$w_i = \frac{f_i}{\max\,(f_j)}, ij \in [synonymous\ codons\ for\ amino\ acid]$$

We calculated the CAI with the group of highly expressed genes used to calculate the ATG context score in the regressor version including features based on expression levels. We achieved a correlation of 0.96 ($p = 10^{-200}$) with the CAI from the yeast DB

(http://www.yeastgenome.org/). In our main regressor scheme which is independent of expression levels, we utilized the regressor train set in order to calculate the CAI. We achieved a correlation of 0.76 ($p = 10^{-200}$) with the CAI from the yeast DB.

The ORF predictor includes the mean CAI across the entire ORF, and the first 100 sliding windows of length 40nt of the ORF. The combined predictor includes these features.

Coding sequence tRNA Adaptation Index (*tAI*), is a statistical model for measuring translational selection. It assumes that the relative concentrations of the tRNA molecules that recognize a codon have a strong effect on the codon translation efficiency. This measure is determined by combining thermodynamic properties of the codon-anticodon interaction, taking into account that due to wobble interactions, several anti-codons can recognize the same codon, with different efficiency weights. The *tAI* [3] gauges the availability of the different tRNA molecules for each codon along an mRNA. The *tAI* is based on the following observation: Since codon-anti-codon coupling is not unique due to wobble interactions, several anti-codons can recognize the same codon, with different efficiency weights.

Let n_i be the number of tRNA isoacceptors recognizing codon i. Let $tCGNij$ be the copy number of the jth tRNA that recognizes the ith codon, and let S_{ij} be a parameter corresponding to the efficiency of the codon-anticodon coupling between codon i and tRNA j. The S_{ij} are inferred optimizing the correlation between the *tAI* and gene expression measurements [3], in the regressor version including features optimized according to expression levels. In our main regressor scheme independent of expression levels, we optimized S_{ij} according to the regressor train set in the following manner. A hill climbing approach was employed by exhaustively permuting across all the potential S values [0.01, 0.25, 0.5, 0.75, 0.95], and selecting those which achieved the best correlation with the CAI, which was calculated as described above.

We define the absolute adaptiveness, W_i , for each codon i as [3, 21]:

$$W_i = \sum_{j=1}^{ni} (1 - S_{ij}) tCGN_{ij}$$

From W_i we obtain w_i, which is the *relative* adaptiveness value of codon i, by normalizing the W_i's values (dividing them by the maximal of all the 61 W_i).

The final *tAI* of a *gene*, *g*, is the following geometric mean:

$$tAIg = \left(\prod_{k=1}^{lg} w_{ikg} \right)^{1/lg}$$

Where i_{kg} is the codon defined by the *k*'th triplet on gene *g*; and *lg* is the length of the gene (excluding stop codons). Thus, the *tAI* of a gene is a number between 0 (extremely non-efficient codons) and 1 (utmost efficiency).

The computation of the *tAI* for the regressor version including features optimized via expression levels is based on tRNA copy numbers, this data was obtained from [21].

Similarly to the CAI, the ORF predictor includes the mean tAI across the entire ORF, and the first 100 sliding windows of length 40nt of the ORF. The combined predictor includes these features.

Totally Asymmetric Exclusion Process (TASEP): The TASEP is a stochastic flow model of translation elongation, whose output is the predicted translation rate, see for example [22, 23] and subsequent studies [24]. In the TASEP, initiation time as well as the time a ribosome spends translating each codon are exponentially distributed with a codon dependent rate. In addition, ribosomes span over several codons and if two ribosomes are adjacent, the trailing one is delayed until the ribosome in front of it has proceeded onwards.

We model an mRNA with *N* codons as a chain of sites, each of which is labeled by *i*. The first and last codons, $i=1$, $i=N$, are associated with the start and stop codons respectively. At any time, *t*, attached to the mRNA are $M(t)$ ribosomes. Being a large complex of molecules, each ribosome will cover *l* codons. Any codon may be covered by a single ribosome or none. To locate a ribosome, we arbitrarily assume that the codon being translated is the one in the middle of the ribosome. For example, if the first, $(l+1)/2$ codons are not covered, a ribosome can bind to the first codon on the mRNA strand, and then it is said to be "on codon $i=1$". A complete specification of the configuration of the mRNA strand is given by the *codon occupation* numbers: $n_i = 1$ if codon *i* is being translated and $n_i = 0$ otherwise. Note that when $n_i = 1$ the $(l-1)/2$ codons before and after codon *i* are covered by the ribosome that is on site *i*, but since they are not the ones being translated the codon occupations numbers for them are equal to zero.

We will now specify the dynamics of the TASEP model. A free ribosome will attach to codon $i=1$ with rate λ, provided that the first $(l+1)/2$ codons on the mRNA are empty. An attached ribosome located at codon i will move to the next codon $i+1$ with rate λ_i, provided codon $i+(l+1)/2$ is not covered by another ribosome. In case $i+(l+1)/2>N$ (ribosome is bulging out of the mRNA strand and cannot be blocked by an additional ribosome) an attached ribosome will move to the next codon with rate λ_i.

In order to simulate these dynamics, we assume that the time between initiation attempts is distributed exponentially with rate λ. Similarly the time between jump attempts from site i to $i+1$ is assumed to be exponentially distributed with rate λ_i. Note that in the case of $i=N$ the jump attempt is in fact a termination step. We define an "event" as an initiation, jump attempt, or termination step. From our definition it follows that the time between events is exponentially distributed (minimum of exponentially distributed random variables) with rate $\mu(\{n_i\}) = \lambda + \sum_{i=1}^{N} n_i \lambda_i$. Note that a jump attempt from codon i can only be made if there is a ribosome translating this codon and hence the rate $\mu(\{n_i\})$ depends on the set of *site occupation* numbers.

The probability that a specific event was an initiation attempt is given by: $\lambda/\mu(\{n_i\})$. Similarly, the probability that a specific event was a jump attempt (or termination event) from site i to site $i+1$ is given by $n_i\lambda_i/\mu(\{n_i\})$.

At each step of the simulation, we determine the nature of the event and the time passed until its occurrence by these rules. The set of *site occupation* numbers are then updated accordingly and the simulation proceeds to the next event. For example if an initiation attempt was made, we check if the first $(l+1)/2$ codons on the mRNA are not covered. If so, we set $n_1 = 1$, otherwise the attempt fails and n_1 remains as is. If a jump attempt from codon i to codon $i+1$ was made, we check if site $i+(l+1)/2$ is not covered. If so, we set $n_i = 0$ and $n_{i+1} = 1$, otherwise the attempt fails and n_i, n_{i+1} remain as is.

Beginning with an empty mRNA strand we simulated the system for 250,000 steps. The system was then simulated for an additional 1,000,000 steps where we kept track of the total number of terminations and the total time that had passed from the point this phase started. The steady state rate of protein production was determined by dividing the number of termination events by the total time that had passed. The number of steps in the second stage was taken after observing that increasing the number of steps fourfold had a negligible effect on the predicted protein production rate.

Codon times are calculated similarly to the *tAI*, for the regressor version including features based on expression levels, and the main regressor scheme independent of expression levels respectively, with the codon times being W.

This feature is relevant only to the ORF and combined predictors.

Evolutionary rates dN, dS and dN/dS: dN/dS is the ratio of the number of non-synonymous substitutions per non-synonymous sites (dN) to the number of synonymous substitutions per synonymous sites (dS), which can be used as an indicator of selective pressure acting on a protein-coding gene. Comparisons of homologous genes with a high dN/dS ratio are usually said to be evolving under positive selection. The estimations for dN, dS, dN/dS were taken from [25]. These three features are relevant only to the ORF and combined predictors, and were utilized only in the regressor version including features optimized via expression levels.

Codon Bias in this context is the frequency of each codon per gene. These 64 features are relevant only to the ORF and combined predictors.

Codon Bias Pairs in this context is the frequency of each codon pair per gene. These 4096 features are relevant only to the ORF and combined predictors.

Amino Acid Bias in this context is the frequency of each amino acid per gene. These 20 features are relevant only to the ORF and combined predictors.

Amino Acid Bias Pairs in this context is the frequency of each amino acid pair per gene. These 400 features are relevant only to the ORF and combined predictors.

Length of the transcript segments, with the combined predictor including in addition to the 5’UTR/ORF/3’UTR lengths, the length of the entire transcript.

The ratio of the length of the 5'UTR to the ORF, this feature is relevant only to the combined predictor.

The ratio of the length of the 3'UTR to the ORF, this feature is relevant only to the combined predictor.

The ratio of the length of the 5'UTR to the 3’UTR, this feature is relevant only to the combined predictor.

Nucleotide composition features

It was previously suggested that the translation elongation step is coupled in various ways with the initiation step via various signals encoded in the codons at the beginning of the coding sequences. For example, it was shown that the first codons of the coding sequences are under selection to induce weak local mRNA folding to improve the efficiency of translation initiation [21, 26, 27]. It was also shown that the codons at the beginning of the coding sequences have lower adaptation to the tRNA pool than the codons afterwards [28], and that the codons ~10-25 downstream from the main ATG are under selection for strong mRNA folding [29], probably to improve ribosomal allocation by decreasing the initiation rate and increasing the distances between ribosomes. In addition, the different parts of the transcripts (5'UTR/ORF/3'UTR) are usually of different length with the ORF usually much longer than the 5'UTR/3'UTR. Thus, it is not clear if the fact that the ORF is a better predictor of gene expression is not simply related to the fact that it is longer than the UTRs. Finally, there may additional signals related to the termination step at the end of the transcript (the 3'UTR).

On this basis, we wanted to investigate the effect of the nucleotide composition of each window of the beginning and end of the coding sequence and its proximal UTR regions, and discern if specific windows/regions have a stronger effect on translation efficiency. To this end we looked at two beginning and end points, expressly 30nt/100nt upstream from the start of the ORF and 200 codons into the ORF, and the last 200 codons of the ORF and 30nt/100nt downstream the end of the ORF. For the transcript segments defined by the proximal 30nt UTR regions, we used sliding windows of length 30nt, and for the 100nt UTR regions windows of length 40nt (30-40nt is the approximated length of the ribosomes' footprint [7]), both with a slide of 1nt. For each position in each window, we calculated 4 binary features representing the appearance (or lack of) of each nucleotide. Each of these 4 binary features represent the appearance of a corresponding nucleotide, A, T, C, G, in position x of window y, such that if for gene *i* an A constitutes that position, the feature representing A in position x of window y would get a 1 at its i*th* index and zero otherwise. The remaining 3 features representing T, C and G for position x of window y, would get a 0 at their i*th* index, as an A had appeared.

Predictors

Transcript sequence predictors

Based on these features we built linear and non linear predictors of the genes sequence segments, the 5'UTR, ORF and 3'UTR separately, and also combining the three together, in the following manner. The data was divided into terciles: a train, test and validation set, performing this sampling 100 times, thus resulting with 100 predictors per segment/entire transcript. This was done in-order to avoid overfitting, and show that there does not exist a single predictor encapsulating the information in each region of the transcript in its entirety,

but much like the nucleotide context of a potential initiating ATG, a statistical distribution of features influencing each stage of gene expression. Additionally, this enabled us to perform statistical analyses of the prevalence, and thus significance of features. We implemented a greedy feature selection process, by which in each iteration every feature is added respectively to the growing regressor, and the feature contributing to the highest correlation is selected, starting with a vector of ones for the linear regressor, and an empty model for the non-linear regressor. At the end of each stage, the current predicted regressor coefficients of the selected features are assessed on the test set. This process is continued, until for 15 not necessarily consecutive iterations, the correlation of the regressor does not improve on the test set (the conclusions reported here are robust to variations in this value). If prior to that threshold being reached the correlation does not improve on the train set, the process is terminated (train set correlations are required to improve in every iteration). The selected regressor is then evaluated on the validation set, in order to avoid overfitting. We used the Spearman rank correlation coefficient, because the Pearson product moment correlation coefficient is strongly biased towards linear trends. Only the Spearman correlation coefficient actually detects a general monotonic trend. Thus, all features were ranked prior to regression analysis.

To model non-linearity we used Multivariate adaptive regression splines (MARS), which is a form of regression analysis introduced in [1]. It is a non-parametric regression technique, and can be seen as an extension of linear models that automatically models non-linearities and interactions. MARS approximates non-linear relationships with continuous piecewise linear functions. The MARS model selects features successively based on their additional contribution to explaining variation in the dependant variable. We employed the Matlab ARESLab implementation of MARS.

For both the linear and non-linear regressors we performed 100 iterations of 3-fold cross-validation. The correlations obtained on the validation set were reported.

Comparison between our approach and the Random-Forest approach

Our approach is similar but not identical to the traditional Random-Forest approach (see, for example [30]). Specifically:

1) In the case of the classical Random-Forest forest a decision tree is inferred while we infer regression models.

2) In each step of building a regressor we (greedily) add one additional feature. In the case of the classical Random-Forest forest a small number of features appear in each node of the decision tree.

3) The traditional Random-Forest approach includes sampling with replacement from the dataset while we performed a similar procedure (Jackknifing).

4) The number of features in each regressor model we study is different, while there are version of the Random-Forest approach where the size of all trees is identical

5) In the case of the traditional Random-Forest eventually a prediction is made based on weighting the predictions of all the trees in the forest; in our case, however, the ranking of features is based on the different models/regressors (number of regressors that include the feature), while we report the performances of the median predictor and not average the predictions of the different predictors.

We believe that a version more similar to random forest should give similar results (similarly to the fact that the non-linear and the linear regressors yield similar performances); however, we haven't checked such versions.

Predictors based on the nucleotide composition of sliding windows across the transcript

We built four types of linear and non-linear nucleotide composition predictors, induced by the above described features. Type 1/2: begins at the last 30/100nt of the 5'UTR, and ends 200 codons into the ORF. Type 3/4: begins at the last 200 codons of the ORF and ends 30/100nt into the 3'UTR. The type 1 and 3 predictors were built according to sliding windows of length 30nt, with a slide of 1nt, each window constituting a predictor, altogether there are 601 such predictors per type. The type 2 and 4 predictors were built according to sliding windows of length 40nt, with a slide of 1nt, each window constituting a predictor, altogether there are 661 such predictors per type. The number of genes participating in each type 1 and 2 predictors is determined by the 5'UTR lengths and ORF lengths, only genes with 5'UTR lengths of at least 30nt and 40nt respectively, and ORF lengths of 200 codons, were included, and thus out of the 4367 genes with known 5'UTRs, 2891 and 1008 genes respectively comprised each predictor. Similarly, the number of genes participating in each type 3 and 4 predictors is determined by the 3'UTR lengths and ORF lengths, only genes with 3'UTR lengths of at least 30nt and 40nt respectively, and ORF lengths of 200 codons, were included, and thus out of the 4367 genes with known 3'UTRs, 3894 and 2140 genes respectively comprised each predictor. The same methodology of greedy feature selection to build the predictors as described in the previous section was employed.

Results of predicting gene expression measurements based on features that may be optimized based on expression levels

Figure S1 includes the results of predictions of gene expression measurements (Figure S1A -- protein levels, Figure S1B -- ribosomal densities, Figure S1C -- mRNA levels) based on the entire transcript when using the combined linear (LIN) predictors (Methods). As can be seen the top features for the protein levels are the transcript PARS exponent, the ORF tAI and the 3'UTR predicted folding energy; the top features for the ribosomal density predictor are the ORF tAI, the transcript length and the ORF TASEP predicted translation rate; and the top

features for mRNA levels predictor are the ORF TASEP predicted translation rate, and the CTT and CGA codons.

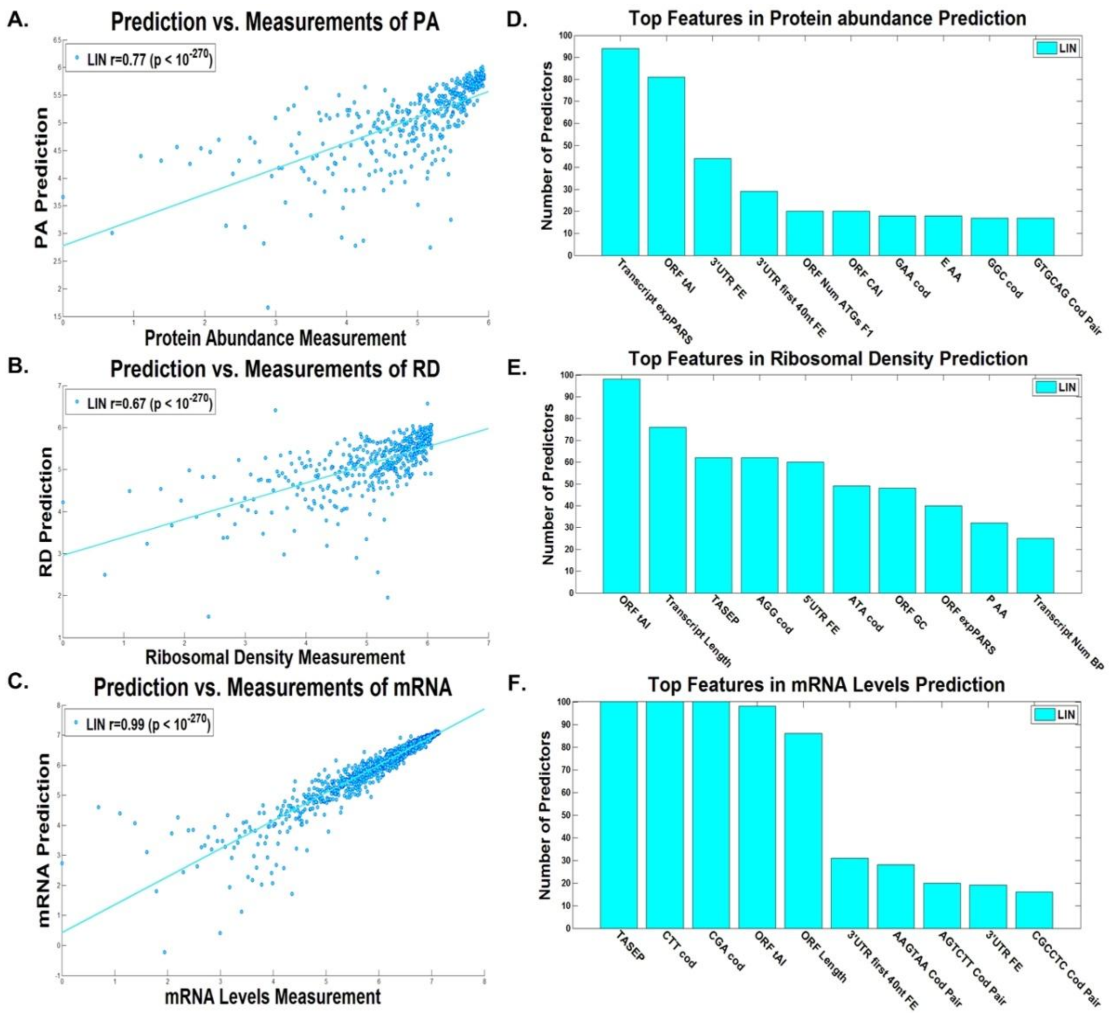

Figure S1: Dot plot of the predictions *vs.* measurements for the validation set of the predictor with the median results for the A. protein levels, B. ribosomal densities, C. mRNA levels, for the entire transcript, i.e. the combined linear (LIN) predictors (Methods). The best features according to the number of predictors they participated in (Methods) of the D. protein levels predictor, E. ribosomal density predictor, and F. mRNA levels predictor, for the entire transcript, *i.e.* the combined linear (LIN) predictors (Methods).

Figure S2 includes the results of predictions of gene expression measurements (Figure S2A -- RL, Figure S2B -- PPR) based on the entire transcript when using the combined linear (LIN) predictors (Methods). As can be seen the top features for the RL predictor are the transcript length, the ORF TASEP predicted translation rate, ORF CAI, and CGA codon; the top features for the PPR predictor are the transcript length and the ORF TASEP predicted translation rate, ORF tAI, and the transcript PARS;

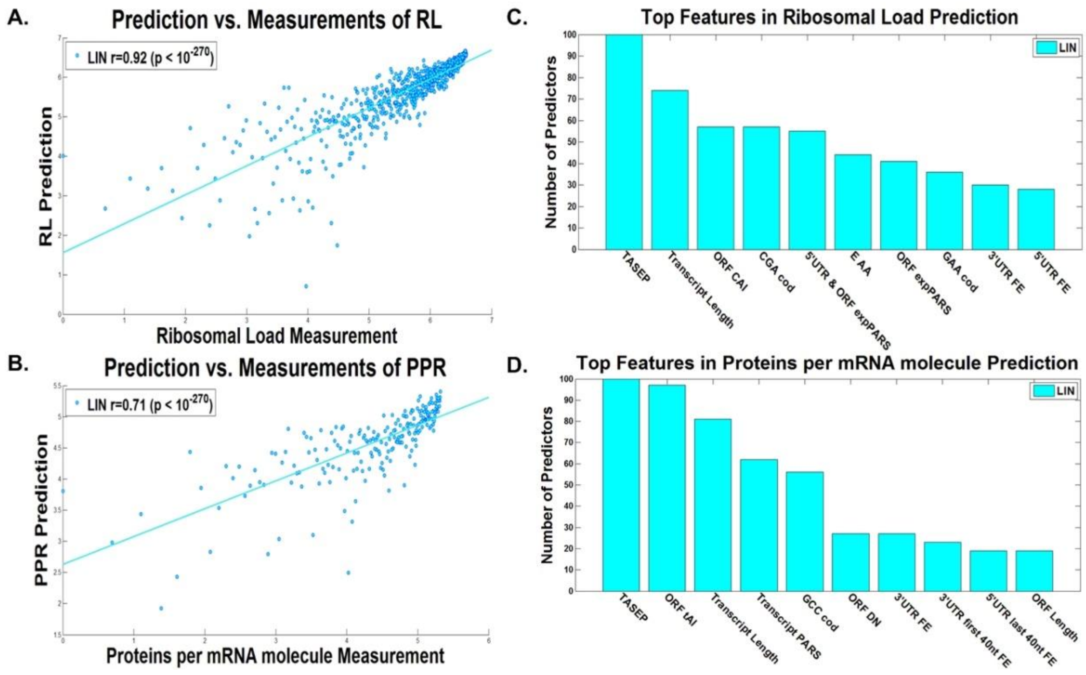

Figure S2: Dot plot of the predictions *vs.* estimated measurements for the validation set of the predictor with the median results for the A. ribosomal load, and B. proteins per mRNA molecule, for the entire transcript, i.e. the combined linear (LIN) predictors (Methods). The best features according to the number of predictors they participated in (Methods) of the C. ribosomal load predictor, and D. proteins per mRNA molecule predictor, for the entire transcript, *i.e.* the combined linear (LIN) predictors (Methods).

Next, we again inferred the five aforementioned predictors (PA, RD, mRNA, RL and PPR) on the basis of the transcript's three main parts *separately*. A summary of the results appears in supplementary Figures S3-S8.

As can be seen the correlations obtained for the linear predictors based on the features of the ORF alone (Figures S5A-C and S6A-B) are very similar to the ones obtained when considering the entire transcript (Figures S1A-C and S2A-B), correlations of 0.71 with PA (based on 17 features on average), 0.66 with RD (20 features on average), 0.99 with mRNA (20 features on average), 0.92 with RL (24 features on average), and 0.69 with PPR (20 features on average) respectively (all p-values < 10^{-130}), suggesting that for inferring a good predictor of endogenous gene expression the information in the UTRs is redundant. The correlations of the linear predictors that are based on the UTRs were markedly lower: correlations of 0.29 with PA (12 features on average), 0.34 with RD (13 features on average), 0.31 with mRNA (12 features on average), 0.39 with RL(14 features on average), and 0.20 with PPR (7 features on average) respectively in the case of the 5'UTR features based predictors (all p-values < 10^{-5}); correlations of 0.25 with PA (13 features on average), 0.33 with RD (18 features on average) , 0.45 with mRNA (20 features on average), 0.53 with RL (21 features on average), and 0.43 with PPR (17 features on average) respectively in the case of the 3'UTR features based predictor (all p-values < 10^{-5}).

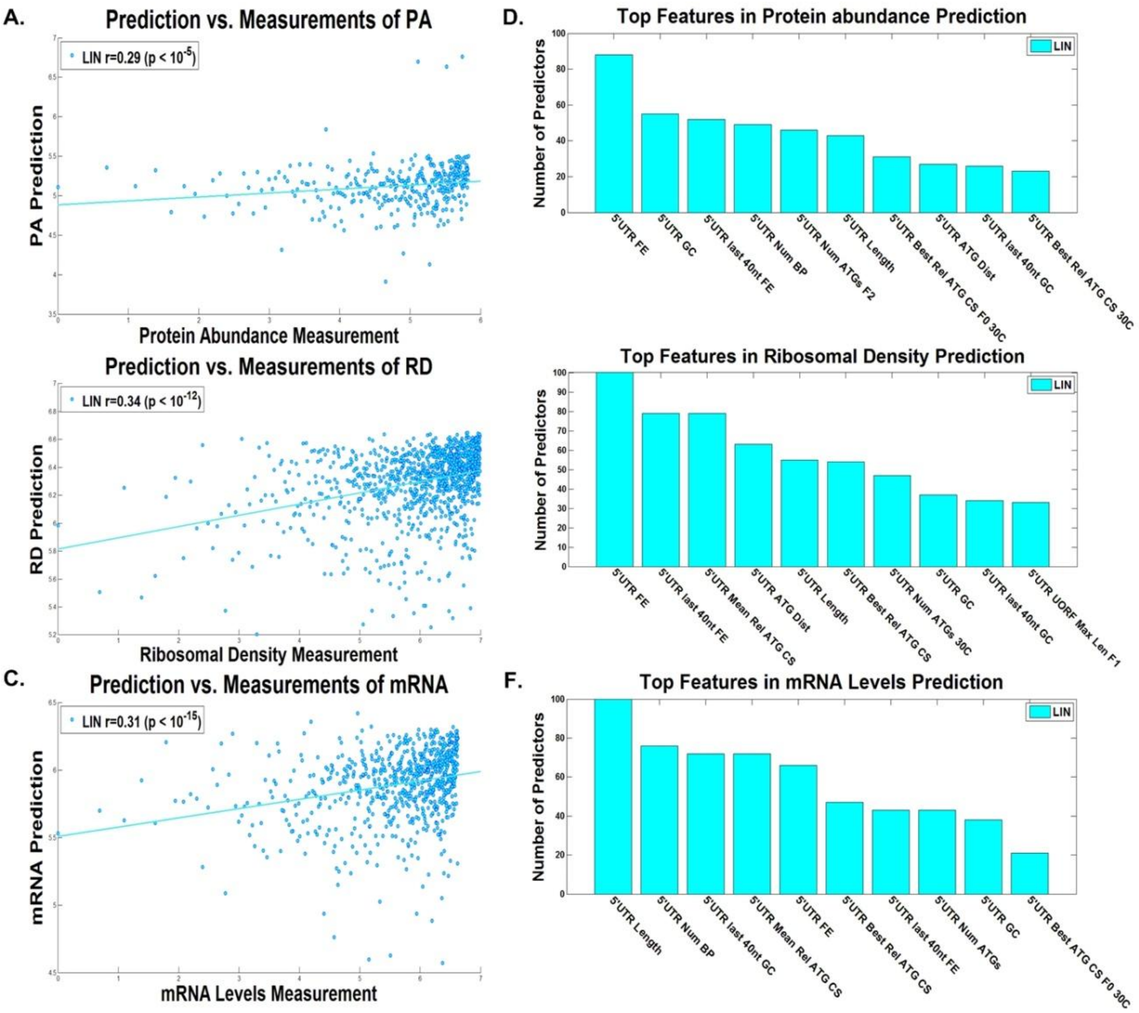

Figure S3: Dot plot of the predictions *vs.* measurements for the validation set of the predictor with the median results for the A. protein levels, B. ribosomal densities, C. mRNA levels, for the 5'UTR linear (LIN) predictors (Methods). The best features according to the number of predictors they participated in (Methods) of the D. protein levels predictor, E. ribosomal density predictor, and F. mRNA levels predictor, for the 5'UTR linear (LIN) predictors (Methods).

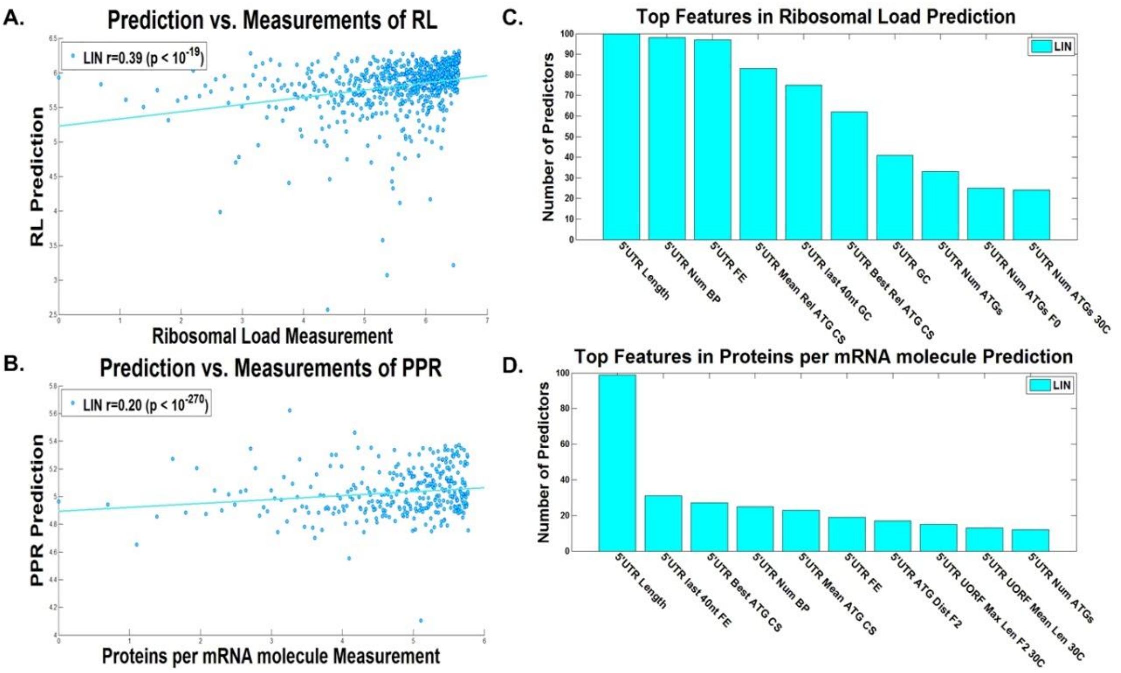

Figure S4: Dot plot of the predictions *vs.* estimated measurements for the validation set of the predictor with the median results for the A. ribosomal load, and B. proteins per mRNA molecule, for the 5'UTR linear (LIN) predictors (Methods). The best features according to the number of predictors they participated in (Methods) of the C. ribosomal load predictor, and D. proteins per mRNA molecule predictor, for the 5'UTR linear (LIN) predictors (Methods).

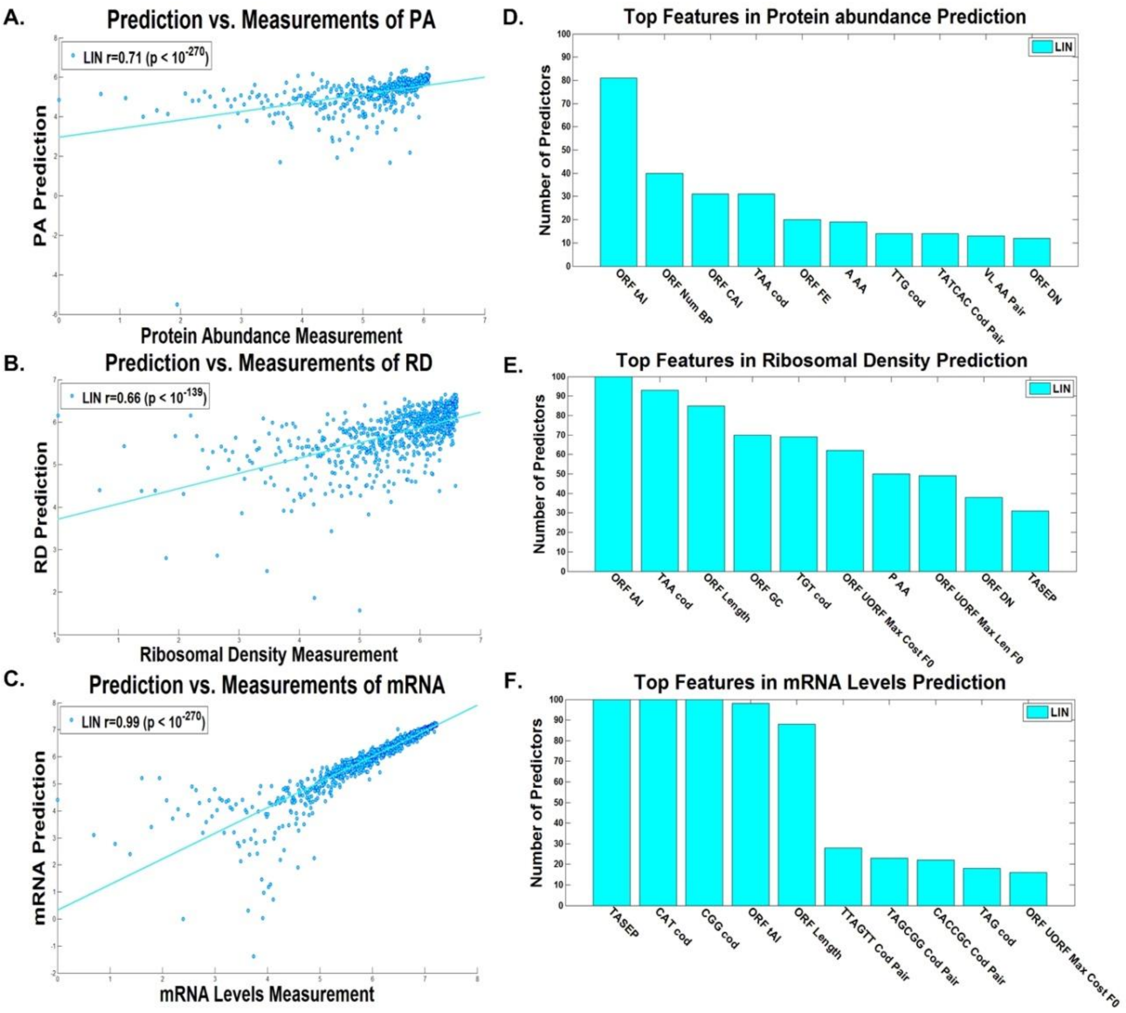

Figure S5: Dot plot of the predictions *vs.* measurements for the validation set of the predictor with the median results for the A. protein levels, B. ribosomal densities, C. mRNA levels, for the ORF linear (LIN) predictors (Methods). The best features according to the number of predictors they participated in (Methods) of the D. protein levels predictor, E. ribosomal density predictor, and F. mRNA levels predictor, for the ORF linear (LIN) predictors (Methods).

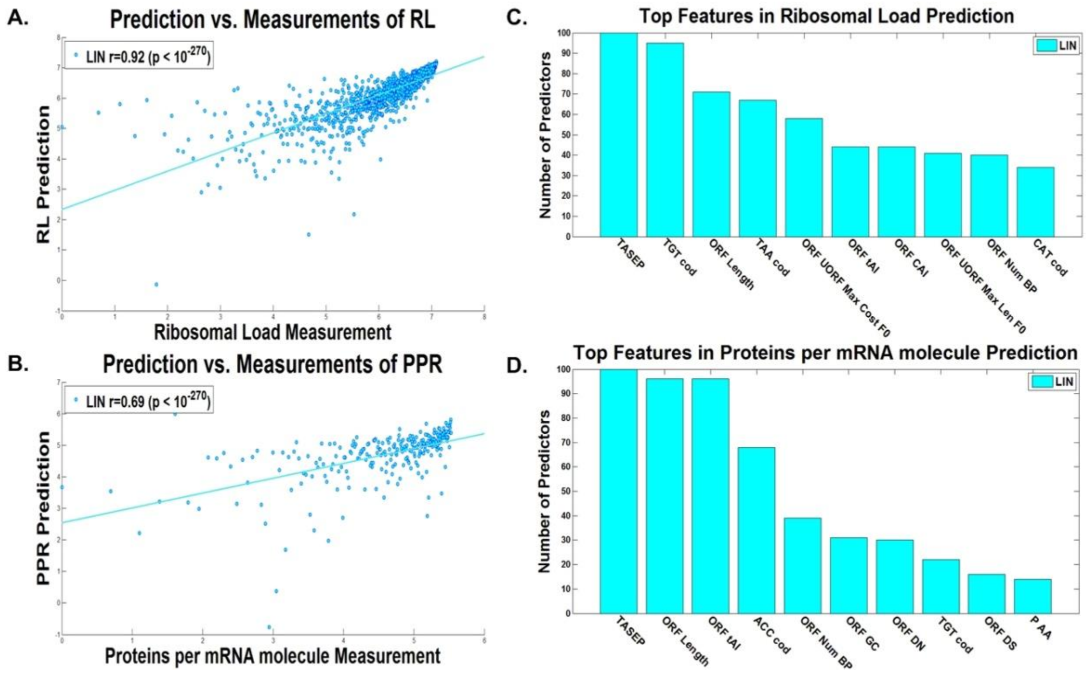

Figure S6: Dot plot of the predictions *vs.* estimated measurements for the validation set of the predictor with the median results for the A. ribosomal load, and B. proteins per mRNA molecule, for the ORF linear (LIN) predictors (Methods). The best features according to the number of predictors they participated in (Methods) of the C. ribosomal load predictor, and D. proteins per mRNA molecule predictor, for the ORF linear (LIN) predictors (Methods).

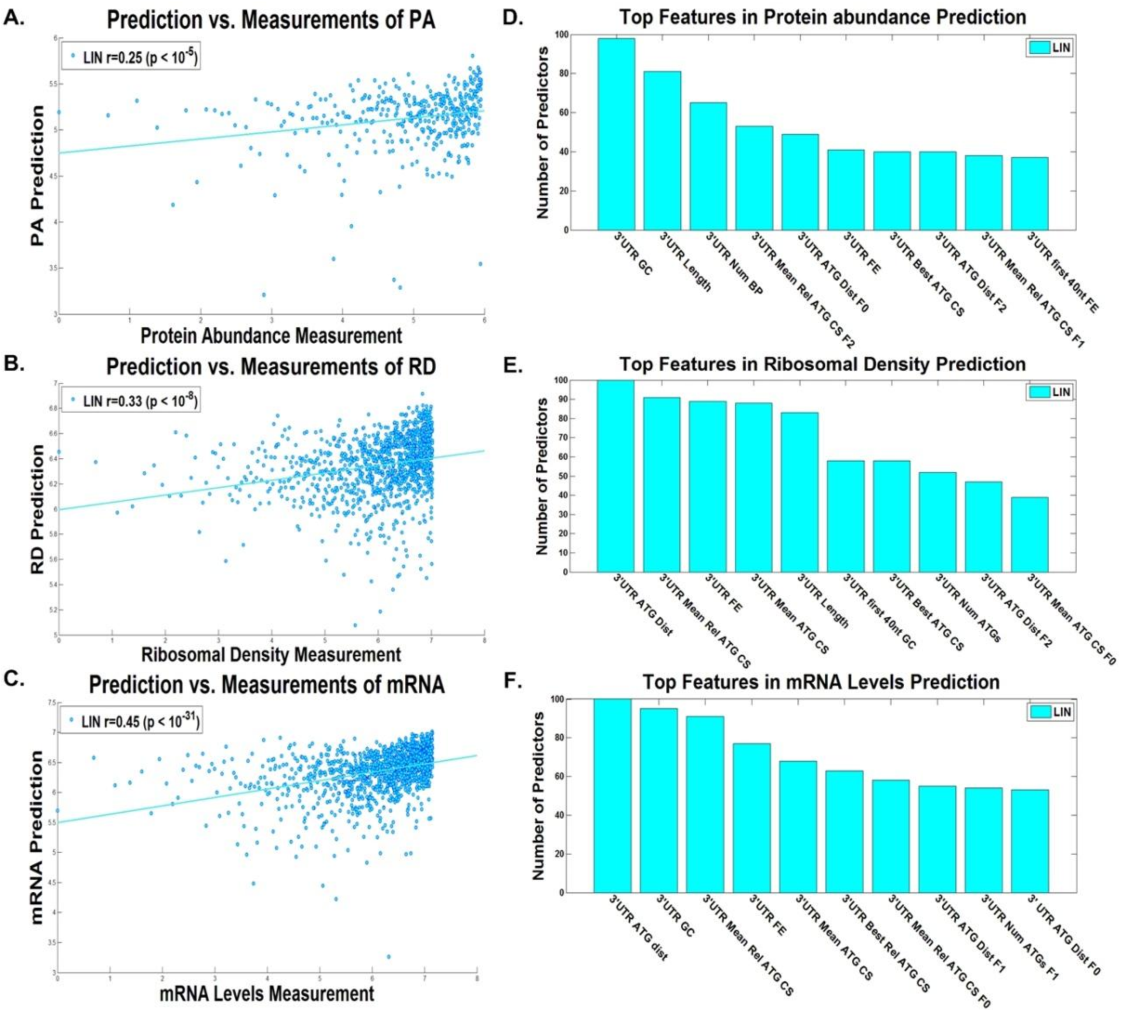

Figure S7: Dot plot of the predictions *vs.* measurements for the validation set of the predictor with the median results for the A. protein levels, B. ribosomal densities, C. mRNA levels, for the 3'UTR linear (LIN) predictors (Methods). The best features according to the number of predictors they participated in (Methods) of the D. protein levels predictor, E. ribosomal density predictor, and F. mRNA levels predictor, for the 3'UTR linear (LIN) predictors (Methods).

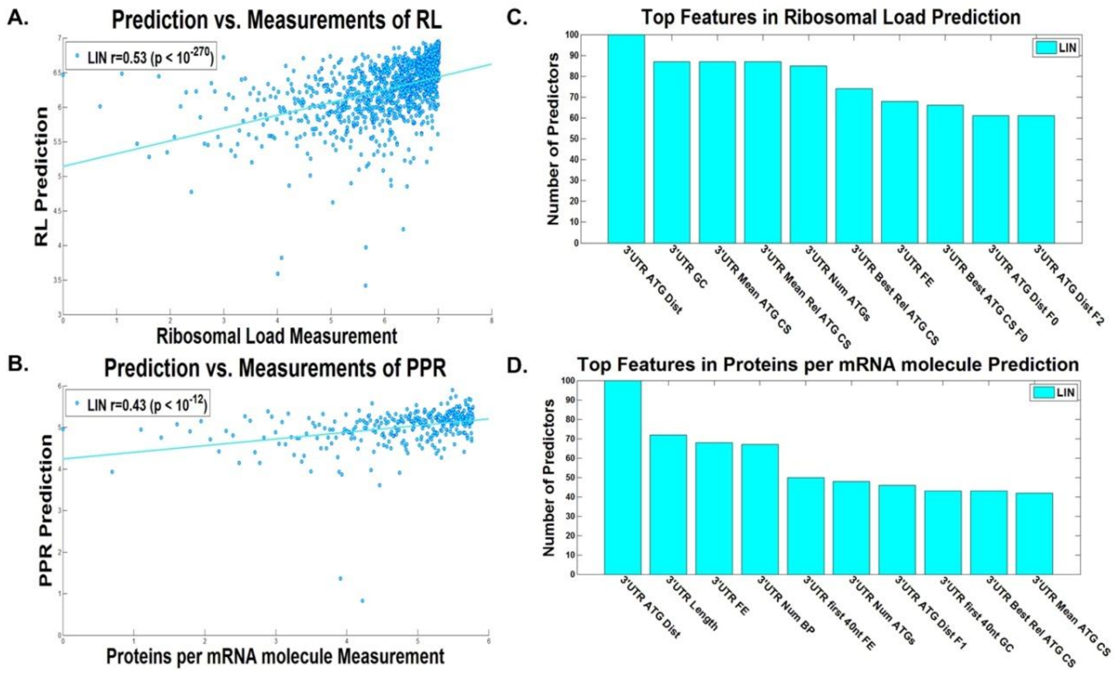

Figure S8: Dot plot of the predictions *vs.* estimated measurements for the validation set of the predictor with the median results for the A. ribosomal load, and B. proteins per mRNA molecule, for the 3'UTR linear (LIN) predictors (Methods). The best features according to the number of predictors they participated in (Methods) of the C. ribosomal load predictor, and D. proteins per mRNA molecule predictor, for the 3'UTR linear (LIN) predictors (Methods)

Predictors based on the nucleotide composition of sliding windows across the transcript

It was previously suggested that the translation elongation step is coupled in various ways with the initiation step via various signals encoded in the codons at the beginning of the coding sequences. For example, it was shown that the first codons of the coding sequences are under selection to induce weak local mRNA folding to improve the efficiency of translation initiation [21, 26, 27]. It was also shown that the codons at the beginning of the coding sequences have lower adaptation to the tRNA pool than the codons afterwards [28], and that the codons ~10-25 downstream from the main ATG are under selection for strong mRNA folding [29], probably to improve ribosomal allocation by decreasing the initiation rate and increasing the distances between ribosomes. In addition, the different parts of the transcripts (5'UTR/ORF/3'UTR) are usually of different length, with the ORF usually much longer than the 5'UTR/3'UTR. Thus, it is not clear if the fact that the ORF is a better predictor of gene expression is not simply related to the fact that it is longer than the UTRs. Finally, there may additional signals related to the translation termination step and mRNA degradation and localization at the end of the transcript (the 3'UTR).

On this basis, we wanted to investigate the effect of the nucleotide composition of each window of the beginning and end of the coding sequence and its proximal UTR regions, and discern if specific windows/regions have a stronger effect on translation efficiency

(Methods). Our initial analysis suggests a rather uniform performance of the predictors along the transcript; thus, it supports the conjecture that the effect of evolution on different parts of the transcript is rather uniform (see Supplementary Figures S9-S12).

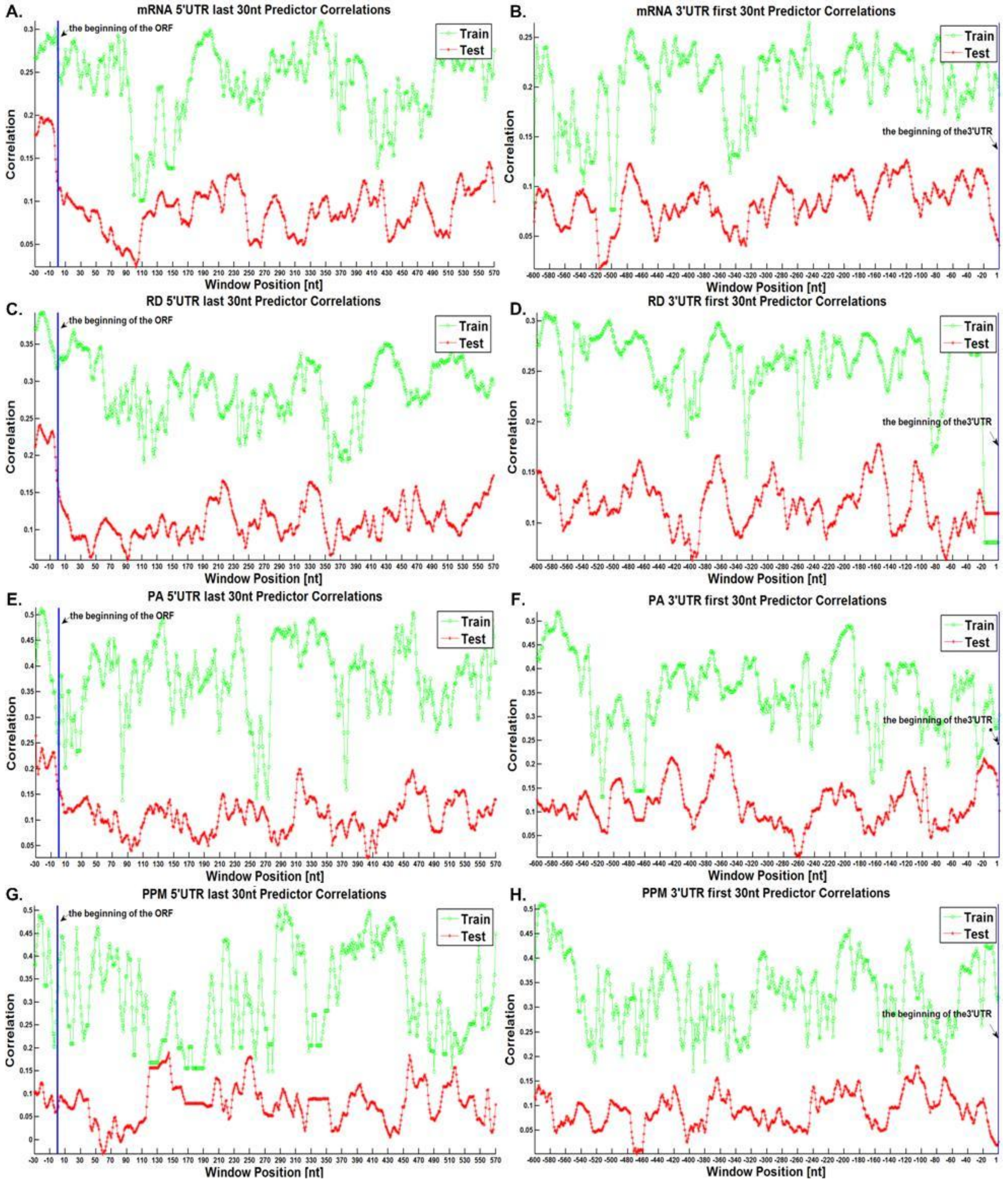

Figure S9: 5’UTR last 30nt into 600nt of the ORF, and last 600nt of ORF into first 30nt of the 3’UTR predictors’ correlations per window position profiles for A. and B. mRNA levels, C. and D. ribosomal densities, E. and F. protein levels, and G. and H. proteins per mRNA molecule.

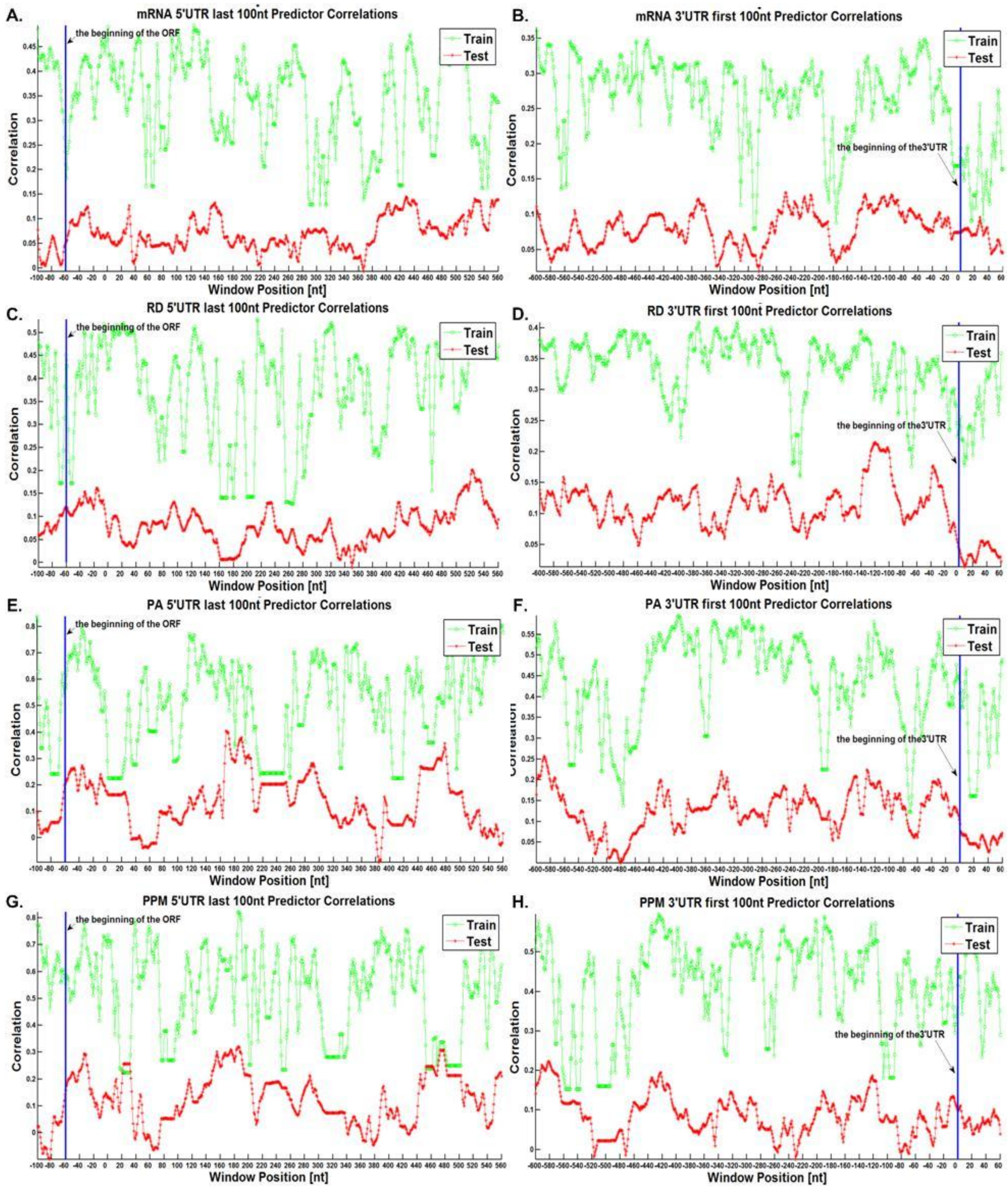

Figure S10: 5'UTR last 100nt into 600nt of the ORF, and last 600nt of ORF into first 100nt of the 3'UTR predictors' correlations per window position profiles for A. and B. mRNA levels, C. and D. ribosomal densities, E. and F. protein levels, and G. and H. proteins per mRNA molecule.

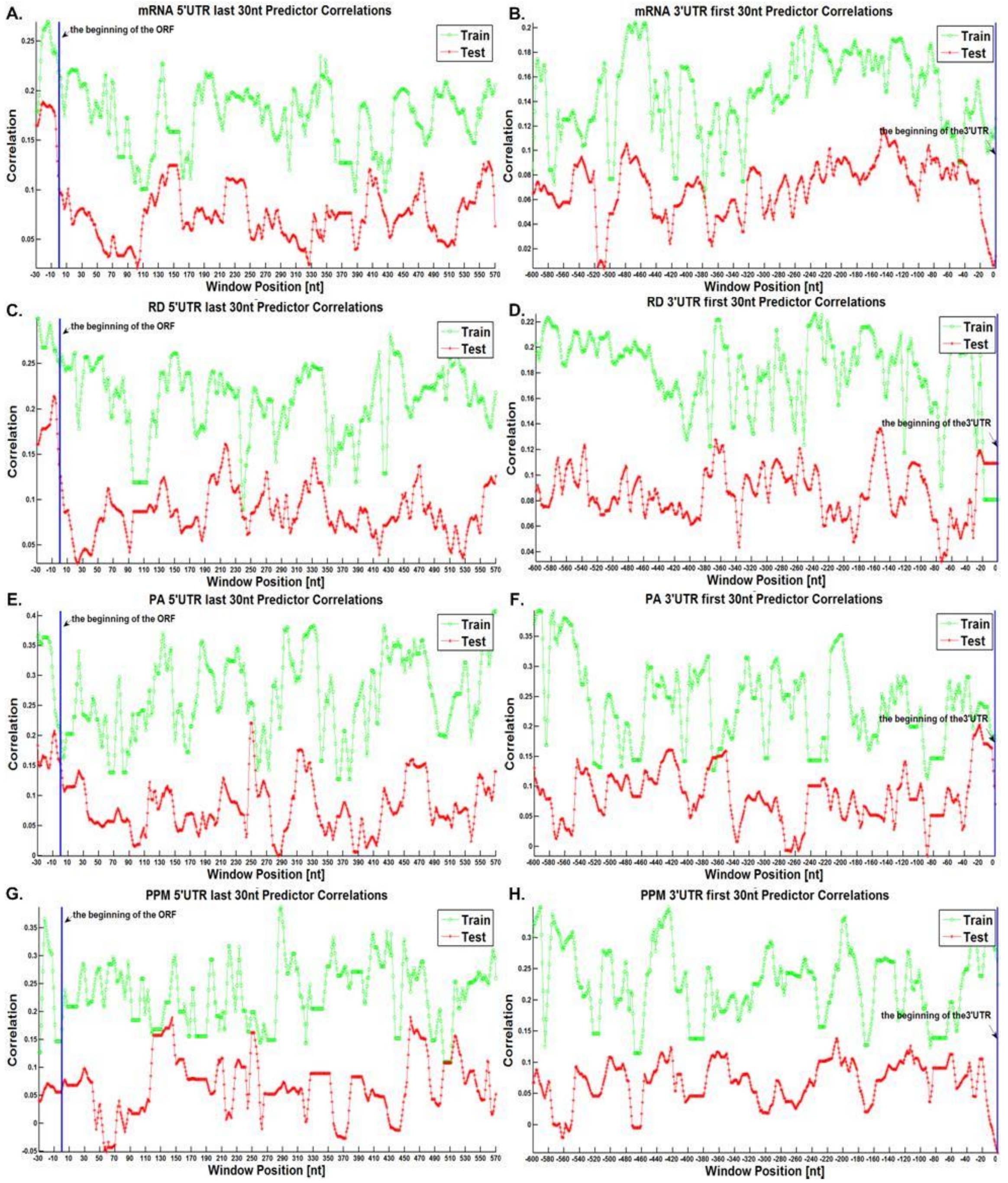

Figure S11: 5'UTR last 30nt into 600nt of the ORF, and last 600nt of ORF into first 30nt of the 3'UTR *non-linear MARS* predictors' (Methods) correlations per window position profiles for A. and B. mRNA levels, C. and D. ribosomal densities, E. and F. protein levels, and G. and H. proteins per mRNA molecule.

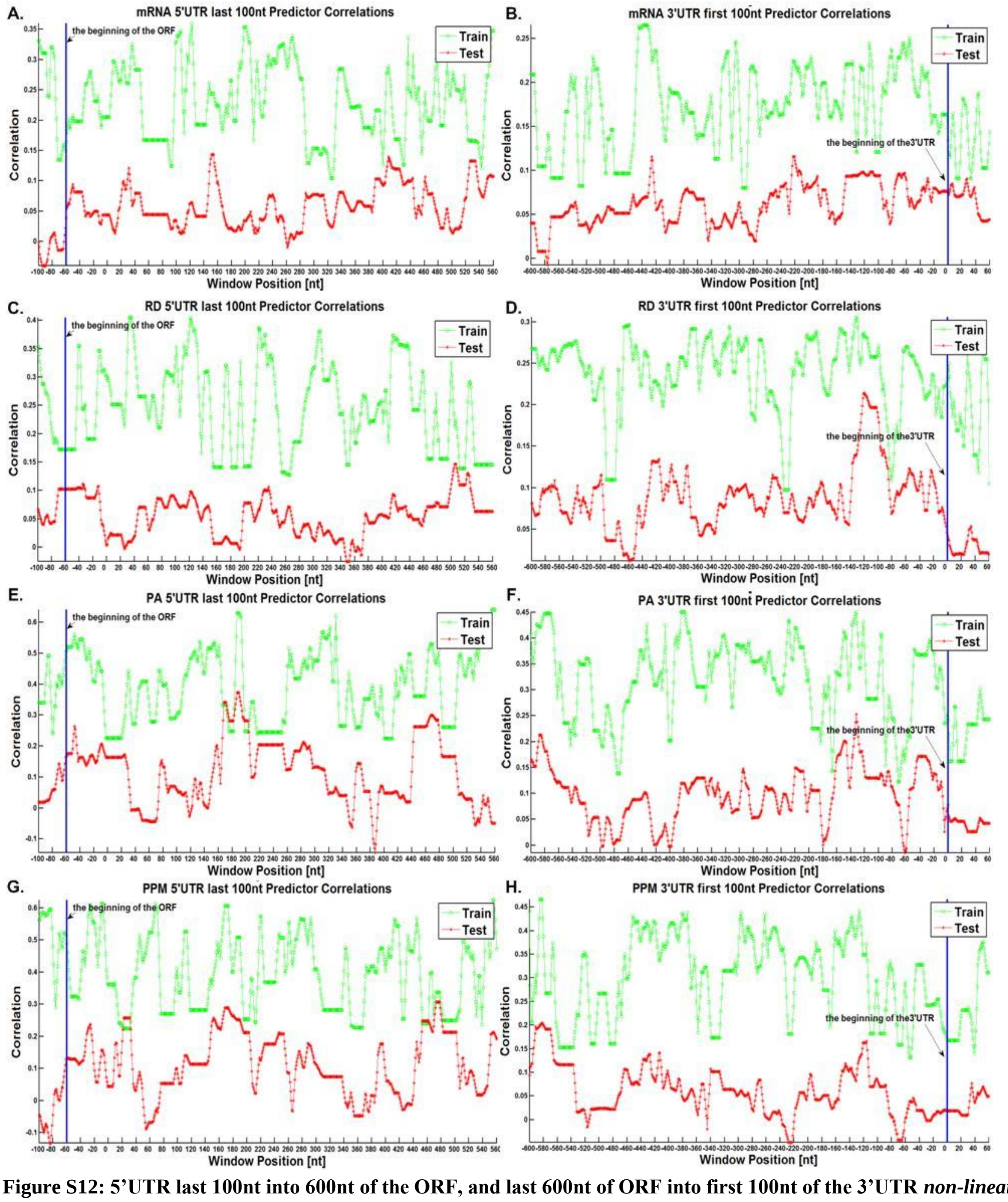

Figure S12: 5'UTR last 100nt into 600nt of the ORF, and last 600nt of ORF into first 100nt of the 3'UTR *non-linear MARS* predictors' (Methods) correlations per window position profiles for A. and B. mRNA levels, C. and D. ribosomal densities, E. and F. protein levels, and G. and H. proteins per mRNA molecule.

References

1. Friedman JH: **Multivariate adaptive regression splines**. *The annals of statistics* 1991:1-67.
2. Sharp PM, Li WH: **The codon adaptation index-a measure of directional synonymous codon usage bias, and its potential applications**. *Nucleic acids research* 1987, **15**(3):1281-1295.
3. dos Reis M, Savva R, Wernisch L: **Solving the riddle of codon usage preferences: a test for translational selection**. *Nucleic acids research* 2004, **32**(17):5036-5044.
4. Pal C, Papp B, Hurst LD: **Highly expressed genes in yeast evolve slowly**. *Genetics* 2001, **158**(2):927-931.
5. Drummond DA, Bloom JD, Adami C, Wilke CO, Arnold FH: **Why highly expressed proteins evolve slowly**. *Proc Natl Acad Sci U S A* 2005, **102**(40):14338-14343.
6. Nagalakshmi U, Wang Z, Waern K, Shou C, Raha D, Gerstein M, Snyder M: **The transcriptional landscape of the yeast genome defined by RNA sequencing**. *Science* 2008, **320**(5881):1344-1349.
7. Ingolia NT, Ghaemmaghami S, Newman JRS, Weissman JS: **Genome-wide analysis in vivo of translation with nucleotide resolution using ribosome profiling**. *Science* 2009, **324**(5924):218.
8. Arava Y, Wang Y, Storey JD, Liu CL, Brown PO, Herschlag D: **Genome-wide analysis of mRNA translation profiles in Saccharomycescerevisiae**. *Proceedings of the National Academy of Sciences of the United States of America* 2003, **100**(7):3889.
9. Ghaemmaghami S, Huh WK, Bower K, Howson RW, Belle A, Dephoure N, O'Shea EK, Weissman JS: **Global analysis of protein expression in yeast**. *Nature* 2003, **425**(6959):737-741.
10. Newman JRS, Ghaemmaghami S, Ihmels J, Breslow DK, Noble M, DeRisi JL, Weissman JS: **Single-cell proteomic analysis of S. cerevisiae reveals the architecture of biological noise**. *Nature* 2006, **441**(7095):840-846.
11. Lee MV, Topper SE, Hubler SL, Hose J, Wenger CD, Coon JJ, Gasch AP: **A dynamic model of proteome changes reveals new roles for transcript alteration in yeast**. *Molecular systems biology* 2011, **7**(1).
12. Wagner A: **Energy constraints on the evolution of gene expression**. *Molecular Biology and Evolution* 2005, **22**(6):1365-1374.
13. Kertesz M, Wan Y, Mazor E, Rinn JL, Nutter RC, Chang HY, Segal E: **Genome-wide measurement of RNA secondary structure in yeast**. *Nature* 2010, **467**(7311):103-107.
14. Zur H, Tuller T: **Strong association between mRNA folding strength and protein abundance in S. cerevisiae**. *EMBO reports* 2012.
15. Alberts B, Johnson A, Lewis J, Raff M, Roberts K, Walter P: **Molecular Biology of the Cell (Garland Science, New York, 2002)**. *b) M Bogyo, M Gaczynska, HL Ploegh, Biopolymers* 2002, **43**:269-280.
16. Jackson R: **Alternative mechanisms of initiating translation of mammalian mRNAs**. *Biochemical Society Transactions* 2005, **33**:1231-1241.
17. Kozak M: **Point mutations define a sequence flanking the AUG initiator codon that modulates translation by eukaryotic ribosomes**. *Cell* 1986, **44**(2):283-292.
18. Kozak M: **Regulation of translation via mRNA structure in prokaryotes and eukaryotes**. *Gene* 2005, **361**:13-37.
19. Zur H, Tuller T: **New Universal Rules of Eukaryotic Translation Initiation Fidelity**. *PLoS Comput Biol* 2013, **in press**.
20. Hamilton R, Watanabe CK, De Boer HA: **Compilation and comparison of the sequence context around the AUG startcodons in Saccharomyces cerevisiae mRNAs**. *Nucleic acids research* 1987, **15**(8):3581-3593.

21. Tuller T, Waldman YY, Kupiec M, Ruppin E: **Translation efficiency is determined by both codon bias and folding energy**. *Proceedings of the National Academy of Sciences* 2010, **107**(8):3645-3650.
22. MacDonald CT, Gibbs JH, Pipkin AC: **Kinetics of biopolymerization on nucleic acid templates**. *Biopolymers* 2004, **6**(1):1-25.
23. Heinrich R, Rapoport TA: **Mathematical modelling of translation of mRNA in eucaryotes; steady states, time-dependent processes and application to reticulocytest**. *Journal of Theoretical Biology* 1980, **86**(2):279-313.
24. Shaw LB, Zia R, Lee KH: **Totally asymmetric exclusion process with extended objects: A model for protein synthesis**. *Physical Review E* 2003, **68**(2):021910.
25. Wall DP, Hirsh AE, Fraser HB, Kumm J, Giaever G, Eisen MB, Feldman MW: **Functional genomic analysis of the rates of protein evolution**. *Proceedings of the National Academy of Sciences of the United States of America* 2005, **102**(15):5483-5488.
26. Kudla G, Murray AW, Tollervey D, Plotkin JB: **Coding-sequence determinants of gene expression in Escherichia coli**. *science* 2009, **324**(5924):255-258.
27. Gu W, Zhou T, Wilke CO: **A universal trend of reduced mRNA stability near the translation-initiation site in prokaryotes and eukaryotes**. *PLoS computational biology* 2010, **6**(2):e1000664.
28. Tuller T, Carmi A, Vestsigian K, Navon S, Dorfan Y, Zaborske J, Pan T, Dahan O, Furman I, Pilpel Y: **An evolutionarily conserved mechanism for controlling the efficiency of protein translation**. *Cell* 2010, **141**(2):344-354.
29. Tuller T, Veksler-Lublinsky I, Gazit N, Kupiec M, Ruppin E, Ziv-Ukelson M: **Composite effects of gene determinants on the translation speed and density of ribosomes**. *Genome biology* 2011, **12**(11):R110.
30. Breiman L: **Random forests**. *Machine learning* 2001, **45**(1):5-32.
